# cAMP receptor protein regulates mouse colonization, motility, fimbria-mediated adhesion, and stress tolerance in uropathogenic *Proteus mirabilis*

**DOI:** 10.1038/s41598-017-07304-7

**Published:** 2017-08-04

**Authors:** Yi-Lin Tsai, Hsiung-Fei Chien, Kuo-Tong Huang, Wen-Yuan Lin, Shwu-Jen Liaw

**Affiliations:** 1 Department and Graduate Institute of Clinical Laboratory Sciences and Medical Biotechnology, College of Medicine, National Taiwan University, Taipei, Taiwan Republic of China; 2 Division of Plastic Surgery, Department of Surgery, Taipei Medical University Hospital and College of Medicine, Taipei Medical University, Taipei, Taiwan Republic of China; 3 Graduate Institute of Toxicology, College of Medicine, National Taiwan University, Taipei, Taiwan Republic of China; 4 Department of Laboratory Medicine, National Taiwan University Hospital, Taipei, Taiwan Republic of China

## Abstract

Cyclic AMP receptor protein (Crp) is a major transcriptional regulator in bacteria. This study demonstrated that Crp affects numerous virulence-related phenotypes, including colonization of mice, motility, fimbria-mediated adhesion, and glucose stress tolerance in uropathogenic *Proteus mirabilis*. Diabetic mice were more susceptible to kidney colonization by wild-type strain than nondiabetic mice, in which the *crp* mutant exhibited increased kidney colonization. Loss of *crp* or addition of 10% glucose increased the *P*. *mirabilis* adhesion to kidney cells. Direct negative regulation of *pmpA* (which encodes the major subunit of P-like fimbriae) expression by Crp was demonstrated using a reporter assay and DNase I footprinting. Moreover, the *pmpA/crp* double mutant exhibited reduced kidney adhesion comparable to that of the *pmpA* mutant, and mouse kidney colonization by the *pmpA* mutant was significantly attenuated. Hence, the upregulation of P-like fimbriae in the *crp* mutant substantially enhanced kidney colonization. Moreover, increased survival in macrophages, increased stress tolerance, RpoS upregulation, and flagellum deficiency leading to immune evasion may promote kidney colonization by the *crp* mutant. This is the first study to elucidate the role of Crp in the virulence of uropathogenic *P*. *mirabilis*, underlying mechanisms, and related therapeutic potential.

## Introduction

Cyclic AMP receptor protein (Crp) is a transcriptional regulator that modulates bacterial metabolism and coordinates the expression of virulence factors through a process known as carbon catabolite repression (CCR), which is triggered in response to the availability of digestible sugars^1–5^. CCR is a common process used by bacteria to optimize their metabolism and enhance their fitness in their natural environments. Therefore, CCR and virulence factor production are often linked in pathogenic bacteria^4^, such as type I fimbriae in *Escherichia coli* and Pla (the plasminogen activator) in *Yersinia pestis*, which causes the pneumonic plague^6, 7^. The mechanisms by which Crp regulates gene expression in response to variable cytoplasmic levels of cAMP have been extensively investigated^1–5, 8^. Crp-regulated genes can be identified by the presence of a Crp box sequence within their promoter regions, and Crp and its cofactor molecular cAMP bind to this sequence to activate or repress gene transcription^1–3^. Crp from several other bacterial species has been characterized and a disparity in Crp regulation was demonstrated among different bacteria; for example, *E*. *coli* Crp represses its own gene expression^9^ in response to glucose^10^, whereas *crp* itself is not autoregulated in *Y*. *pestis*. Instead, *Y*. *pestis crp* is regulated by the PhoPQ two-component system (TCS)^11^.

*Proteus mirabilis* is a common opportunistic urinary tract pathogen, particularly in patients with indwelling catheters^12^. Several virulence factors of *P*. *mirabilis* have been demonstrated to contribute to infections, including fimbriae, flagella, hemolysin, urease, and ZapA metalloprotease^12–14^. Mannose-resistant *Proteus*-like (MR/P) fimbriae were synthesized *in vivo* and implicated in adherence and biofilm formation^15^. P pili (or P fimbriae) were shown to be highly associated with acute pyelonephritis caused by uropathogenic *E*. *coli* (UPEC)^16^. The pyelonephritis-associated pili (*pap*) gene cluster encodes the proteins required for the biogenesis of P fimbriae. Notably, the *P*. *m* *irabilis* P (*pmp*) fimbria-like gene cluster was found in almost all uropathogenic *P*. *mirabilis* species^17^. In addition, *P*. *mirabilis* exhibits a form of multicellular behavior termed swarming^13^. The regulation of swarming motility depends on several factors, including the flagellar master regulator FlhDC^12^ and specific nutrients and environmental cues, such as amino acids (L-arginine and L-glutamine)^18^ and some carbohydrates^19^. In this regard, Crp mediates glucose repression of the swarming associated *sdh* operon^19, 20^. Because Crp-cAMP binds to the *flhDC* promoter region and interacts with RNA polymerase to activate transcription, the cAMP-dependent catabolite repression systems of *E*. *coli* and *Serratia marcescens* are required for the production of flagella and flagellum-based motility^21, 22^.

Urinary tract infections (UTIs) are common in patients with diabetes, with associated complications such as emphysematous cystitis and pyelonephritis if not properly managed^23–25^. Poor glycemic control, various immune system impairments, and bladder dysfunction unique to diabetes may increase the risk of UTIs in these patients^23, 24, 26^. In addition, higher glucose concentrations in urine may promote the growth of pathogenic bacteria. Notably, *Proteus* species are among the most common pathogens isolated from the urine of diabetic patients with UTIs^24, 27, 28^. However, the reasons for this high incidence have not been thoroughly investigated.

In this study, we investigated the roles of Crp in uropathogenic *P*. *mirabilis* by creating a *crp* mutant and using different glucose conditions to mimic alterations in Crp activity. We observed that *crp* expression and Crp activity affected numerous virulence-related phenotypes, including urinary tract colonization of mice, motility, fimbria-mediated adhesion, and stress tolerance. In addition, the relationship of Crp with the glucose PTS transporter (PtsG)^10, 29^, adenylate cyclase (CyaA)^30^, or RNA chaperone (Hfq)^31^ was investigated. Hyperglycemia is a hallmark of patients with diabetes, and excessive glucose in the urine and/or blood is generally considered to reduce the ability of immune cells to break down bacteria^26^. However, few studies have investigated the role of the virulence factors of pathogens in UTIs in hosts with diabetes. Hence, we also created a hyperglycemic mouse model to investigate the influence of Crp on urinary tract colonization. Diabetic mice exhibited significantly increased kidney colonization (*p* < 0.01) by the wild-type *P*. *mirabilis* rather than the *crp* mutant compared with nondiabetic mice, which implies that Crp plays a role in kidney colonization in diabetic mice. Crp-mediated upregulation of P-like fimbriae, increased survival in macrophages, increased stress tolerance, upregulation of RpoS, and flagellum deficiency (leading to immune evasion) caused by *crp* loss or reduced Crp activity may promote mouse kidney colonization. This is the first study to elucidate the Crp-regulated virulence factors of uropathogenic *P*. *mirabilis*, underlying mechanisms, and related therapeutic potential. Moreover, this study provides new insights into the role of *P*. *mirabilis* Crp in urinary tract colonization of a host with diabetes.

## Results

### Identification and characterization of *P*. *mirabilis crp*

**Figure 1:**
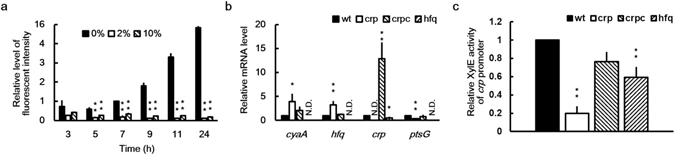
Crp activity was inhibited by high glucose levels; *cyaA*, *hfq*, *ptsG*, and *crp* itself were regulated by Crp, and *crp* was regulated by Hfq in *P*. *mirabilis*. (**a**) Crp activity increased in the absence of glucose and decreased by 2% and 10% (2000 and 10,000 mg/dL) glucose. The *plac-gfpuv* reporter plasmid-transformed wild-type *P*. *mirabilis* was grown in LB broth with or without glucose, and the fluorescent intensity of GFPuv was monitored after incubation for 3, 5, 7, 9, 11, and 24 h. The fluorescent intensity in the absence of glucose at 7 h was set at 1, and all other data are expressed relative to this value after being normalized against the OD_600_ value. The data are the averages and standard deviations of three independent experiments. The significant difference between the presence and absence of glucose at each time point was determined using the Student’s *t* test (**P < 0.01). (**b**) Crp regulated the expression of *cyaA*, *hfq*, and *ptsG* mRNA, and *crp* expression was regulated by Hfq in *P*. *mirabilis*. Overnight bacterial cultures were diluted in LB broth and incubated for 7 h before total RNA was prepared for measuring mRNA levels of *cyaA*, *hfq*, *crp*, and *ptsG* in the wild-type strain, *crp* mutant, *crp*-complemented strain, or *hfq* mutant by using real-time RT-PCR. The mRNA level for the wild-type strain was set at 1, and all other data are expressed relative to this value. N.D., not determined. (**c**) *crp* promoter activities of wild-type *P*. *mirabilis*, *crp* mutant, *crp*-complemented strain, and *hfq* mutant. The activities of XylE in the *crp-xylE* reporter plasmid-transformed *P*. *mirabilis* strains were determined using the reporter assay at 7 h after incubation. The value obtained for the wild-type strain was set at 1. In (**b**) and (**c**), the data are the averages and standard deviations of three independent experiments. Significant difference from the wild-type strain is indicated with an asterisk (*P < 0.05; **P < 0.01 by using Student’s *t* test). wt, wild-type; crp, *crp* mutant; crpc, *crp*-complemented strain; hfq, *hfq* mutant.

As shown in Supplementary Fig. S1a, *crp* was identified from nucleotides 3093805–3094437 in the genome of *P*. *mirabilis* strain HI4320. It is located between *PMI2819* and *argD*. *P*. *mirabilis* Crp comprises 210 amino acids and shares 98–99% sequence identity with *E*. *coli* MG1655, *Salmonellla enterica* Typhimurium LT2, *Y*. *pestis* CO92, and *S*. *marcescens* Db11. Using primers annealing to conserved sequences, we cloned and sequenced the DNA fragment containing *crp* and its upstream region of *P*. *mirabilis* N2. The Crp amino acid sequence of *P*. *mirabilis* N2 was 100% identical to that of *P*. *mirabilis* HI4320. Because *E*. *coli* Crp is activated in response to low glucose (LB broth), a condition that activates CyaA for the production of cAMP^10^, we monitored the expression of the *plac-gfpuv* reporter, which is regulated by Crp-cAMP at 3, 5, 7, 9, 11 and 24 h in wild-type *P*. *mirabilis* after exposure to 0%, 2%, and 10% glucose. Crp activity increased over time in the absence of glucose, but was inhibited in the presence of 2% or 10% glucose (Fig. 1a). Crp has been described as a master regulator, activating or repressing the expression of many genes in *E*. *coli* ^3^. In contrast to *ptsG* activation^29^, *E*. *coli* Crp-cAMP negatively regulates the expression of *cyaA* (adenylate cyclase gene)^32^ and *hfq* ^31^. Notably, *Y*. *pestis* Hfq exhibits specific transcriptional and posttranscriptional regulation of Crp^7^. To investigate the role of Crp, we generated an isogenic mutant lacking the entire *crp* coding sequence. We monitored the growth of the wild-type strain, *crp* mutant, and *crp*-complemented strain (crpc) in LB broth. A growth defect was detected in the *crp* mutant compared with the wild-type and crpc strains (Supplementary Fig. S1d). Overnight cultures of the *crp* mutant routinely reached a density at 600 nm of 0.6–0.7 rather than 1 for the wild-type strain.

To investigate the effect of *crp* loss on the expression of *cyaA*, *hfq*, and *ptsG*, we assessed the mRNA levels of *cyaA*, *hfq*, and *ptsG* by using real-time reverse transcription polymerase chain reactions (RT-PCRs) in wild-type *P*. *mirabilis*, *crp* mutant, and crpc strains. We observed that Crp positively regulated the expression of *ptsG* mRNA and negatively regulated that of *hfq* and *cyaA* (Fig. 1b). The XylE reporter assay revealed a positive autoregulation in *crp* itself (Fig. 1c). In addition, we observed that the loss of *hfq* reduced both the mRNA level (Fig. 1b) and promoter activity (Fig. 1c) of *crp*. Collectively, these findings imply that the expression of *P*. *mirabilis ptsG*, *cyaA*, and *hfq* can be modulated by Crp-cAMP under normal LB culture conditions. Moreover, Crp is positively autoregulated and also upregulated by Hfq.

The loss of *crp* has been demonstrated to have metabolic defects in some bacterial species^20, 33, 34^. Accordingly, a metabolic analysis with the VITEK GN ID card (bioMérieux) revealed that the *P*. *mirabilis crp* mutant had defects in citrate utilization, trehalose fermentation, succinate alkalinization, and activities of ornithine decarboxylase and γ-glutamyl transferase (data not shown). Furthermore, we used a transcriptome to compare the gene expression of wild-type *P*. *mirabilis* in the presence of 10% glucose (wt-10% glc) and the *crp* mutant. Total RNA was extracted and subjected to differential RNA-sequencing (RNA-seq) analysis^35^. Supplementary Fig. S1e presents a scattergram of the gene expression of the 10% glucose-treated wild-type strain (x-axis) and *crp* mutant (y-axis) relative to the wild-type strain (log_2_ fold-change values). Among 3217 genes detected in wild-type *P*. *mirabilis* by using RNA-seq, 1253 genes were upregulated (quadrant I) and 628 genes were downregulated (quadrant III) in both the 10% glucose-treated wild-type strain and *crp* mutant. A correlation (up to 60%) was observed between the gene expression profiles of the 10% glucose-treated wild-type strain and *crp* mutant. In other words, an up to 60% correlation of gene expression profiles between the 10% glucose-treated wild-type strain and *crp* mutant was observed, and more than 40% of the genes were expressed in an opposite manner between them. When more than twofold selection was applied to the expression change, 202 genes were upregulated (quadrant I) and 211 genes were downregulated (quadrant III) in both the 10% glucose-treated wild-type strain and *crp* mutant. Moreover, 64 genes exhibited an opposite expression trend between the 10% glucose-treated wild-type strain and the *crp* mutant both in quadrants II and IV (Supplementary Excel file).

### The *crp* mutant exhibited an increased ability to colonize the kidneys of nondiabetic mice, and Crp might play a role in kidney colonization in diabetic mice

**Figure 2:**
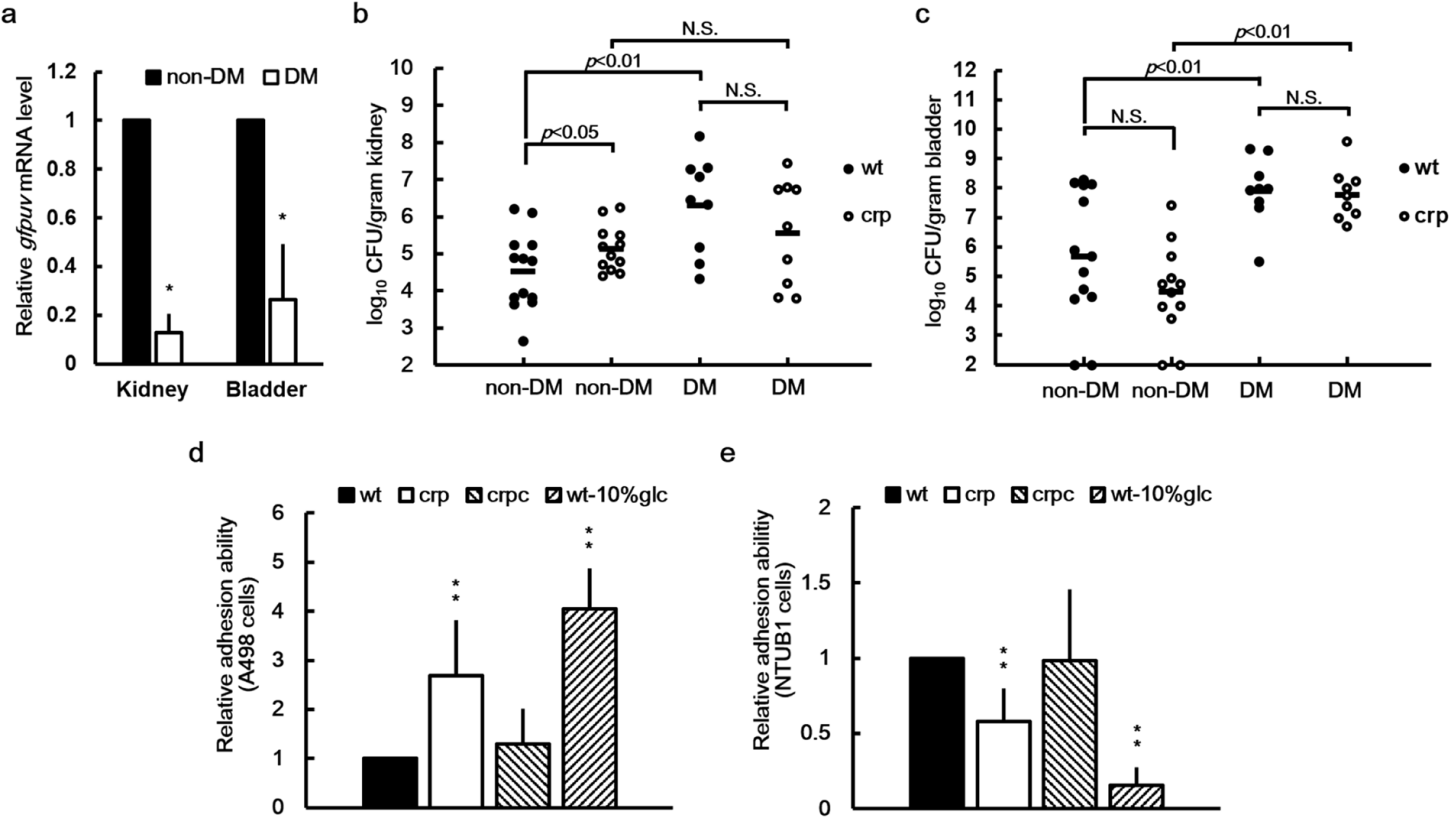
Crp activity, urinary tract colonization, and urothelial cell adhesion of *P*. *mirabilis* were affected by the diabetic environment, loss of *crp* or the high glucose level. (**a**) Crp activity of *P*. *mirabilis* was decreased in the kidneys and bladders of diabetic mice. Six-week-old female mice were injected transurethrally with overnight cultures of the *plac-gfpuv* reporter plasmid-transformed wild-type *P*. *mirabilis* strains (10^7^ CFU per mouse). On day 3 after injection, the mice were sacrificed, and bladder and kidney samples were collected to quantify the mRNA content of *gfpuv* using real-time RT-PCR. The mRNA level for each organ of nondiabetic mice was set at 1. The data are the averages and standard deviations of three independent experiments. The significant difference between nondiabetic mice (non-DM) and diabetic mice (DM) was observed using Student’s *t* test (*P < 0.05). Kidney (**b**) and bladder (**c**) colonization in DM and non-DM by wild-type *P*. *mirabilis* (wt) or *crp* mutant (crp). Diabetic or nondiabetic ICR mice (at least nine mice per group) were inoculated transurethrally with overnight cultures of bacteria at a dose of 10^7^ CFU per mouse. Bacterial loads (CFU) in the kidneys and bladders were determined on day 3 after inoculation. Horizontal bars indicate the average for each group, and the limit of detection was 100 CFU/g organ. Significant differences were determined using the Mann–Whitney test, except for the difference between colonization by the wild-type strain and *crp* mutant in the kidneys of nondiabetic mice as determined using the Student’s *t* test. N.S., no significant difference. Adhesion of wild-type in the absence (wt) or presence of 10% glucose (wt-10%glc), the *crp* mutant (crp), and the *crp*-complemented strain (crpc) to kidney (**d**) and bladder (**e**) epithelial cells. Adhesion abilities to A498 cells and NTUB1 cells were determined as described in the Materials and Methods section. The adhesion ability of the wild-type strain was set at 1 and other data are relative to this value. The data represent the averages and standard deviations of three independent experiments. The significant difference from the wild-type strain is indicated with an asterisk (**P < 0.01, using Student’s *t* test).

*Proteus* spp. are among the most common pathogens in diabetic patients with UTIs^24, 27, 28^. Because Crp is often linked to virulence factor production in pathogenic bacteria^4–7, 36^ and because its activity is associated with glucose levels, we hypothesized that *P*. *mirabilis* Crp-cAMP (correlated with glucose levels) controls the expression of virulence factors for establishing UTIs in the diabetic host. Therefore, we studied streptozotocin-induced diabetic ICR mice, whose blood and urine glucose levels were approximately 440.17 and more than 1000 mg/dL, respectively (approximately 152 and less than 50 mg/dL for nondiabetic mice, respectively). First, we tested whether high-glucose conditions alter *P*. *mirabilis* Crp activity in diabetic mice. The mice were injected transurethrally with wild-type *P*. *mirabilis* harboring the *plac-gfpuv* reporter plasmid, and the *gfpuv* mRNA levels of *P*. *mirabilis* in the bladders and kidneys were measured. The results indicated that the Crp activity (shown as the *gfpuv* mRNA level) of *P*. *mirabilis* in diabetic mice significantly decreased relative to that in nondiabetic mice (Fig. 2a). Thus, *P*. *mirabilis* Crp activity was modulated by high glucose *in vivo* and could be involved in UTIs. Subsequently, we injected diabetic and nondiabetic ICR mice with wild-type *P*. *mirabilis* and the *crp* mutant, respectively, to examine the effect of *crp* loss on the urinary tract colonization of mice. On day 3 after inoculation, the mice were sacrificed to collect kidney and bladder samples, and the samples were homogenized to determine the viable bacterial count. Diabetic mice were more susceptible to kidney and bladder colonization by wild-type *P*. *mirabilis* than nondiabetic mice (Fig. 2b and c). The loss of *crp* abolished this phenomenon in kidneys but not in bladders. Notably, *crp* mutant colonization of nondiabetic mice significantly increased in the kidneys (*p* < 0.05) (although the difference was small) and nonsignificantly decreased in the bladder, compared to the wild-type strain. No difference in the ability to colonize the bladders and kidneys of diabetic mice was observed between the wild-type strain and the *crp* mutant, possibly due to the downregulation of Crp activity under high-glucose conditions in diabetic mice. In summary, *P*. *mirabilis crp* expression and Crp activity were involved in kidney colonization in diabetic mice, and the loss of *crp* increased kidney colonization in nondiabetic mice. We then investigated the underlying mechanisms.

### Loss of *crp* or high glucose reduced swarming, production of flagellin/flagella, and *flhDC* expression in *P*. *mirabilis*

**Figure 3:**
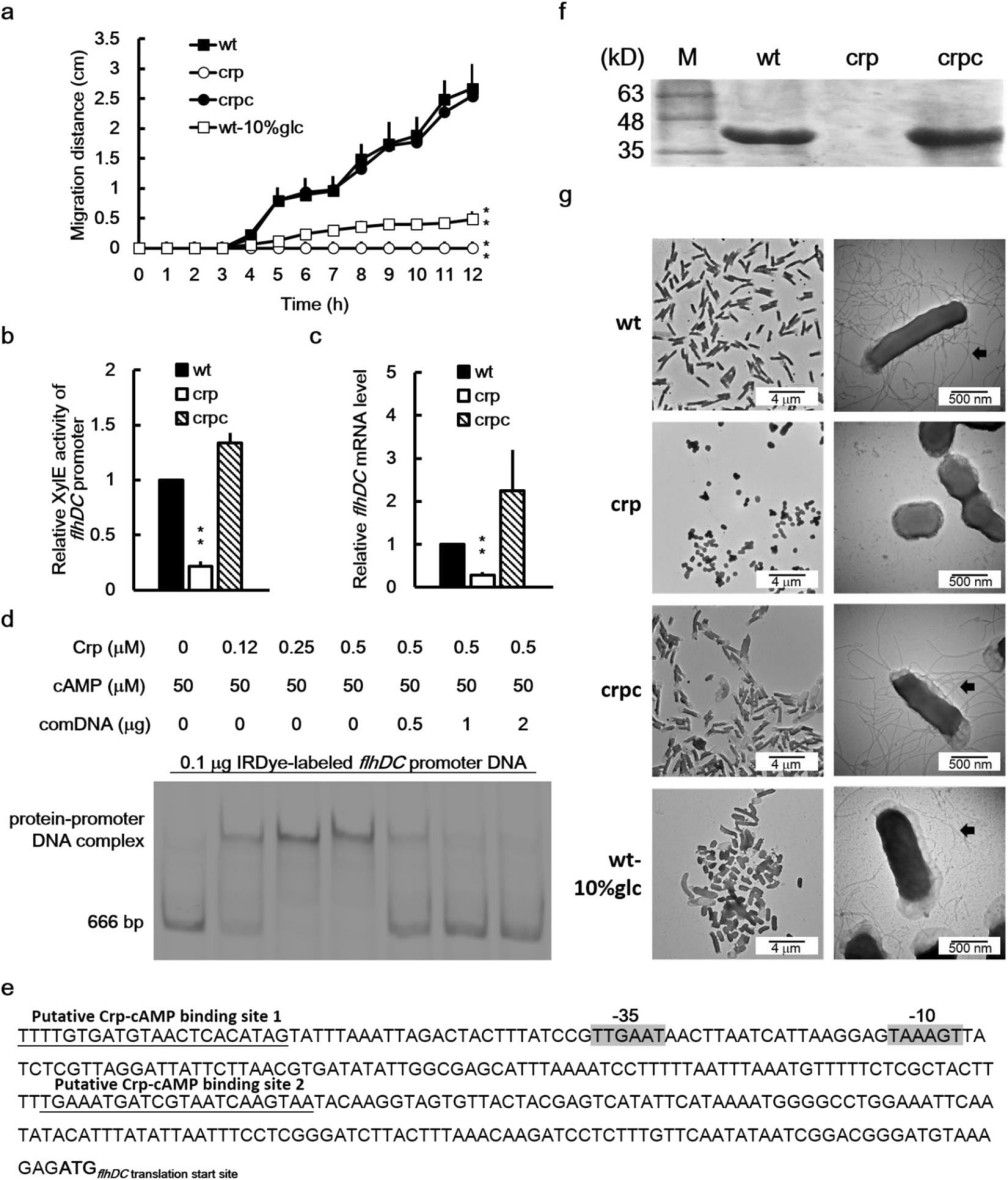
Loss of *crp* or high glucose reduced swarming, production of flagellin/flagella and *flhDC* expression in *P*. *mirabilis*. **(a)** Swarming migration of the wild-type in the absence or presence of 10% glucose, *crp* mutant and the *crp*-complemented strain. An aliquot (5 μl) of overnight cultures was inoculated centrally onto the swarming plate. The migration distance was measured hourly after inoculation. The significant difference from wild-type is indicated with an asterisk at 12 h. (**b**) Promoter activities of *flhDC* in wild-type, *crp* mutant, and *crp*-complemented strain. The activities of XylE in the *flhDC-xylE* reporter plasmid-transformed bacterial strains were determined using the reporter assay at 7 h after incubation. The value obtained for the wild-type was set at 1. (**c**) The *flhDC* mRNA levels in the wild-type, *crp* mutant, and *crp*-complemented strain. The *flhDC* mRNA amounts were quantified using real-time RT-PCR at 7 h after incubation. The value obtained for the wild-type was set at 1. In (**a**),(**b**) or (**c**), the data represent the averages and standard deviations of three independent experiments. Significant difference from the wild-type is indicated with an asterisk (**P < 0.01 by Student’s *t* test). (**d**) The binding of *P*. *mirabilis* Crp-cAMP to the *flhDC* promoter region revealed using an EMSA. IRDye-labeled DNA fragments (0.1 μg) of the *flhDC* promoter region (666 bp) obtained by PCR were incubated with the purified His-tagged Crp (0–0.5 μM) in the presence of cAMP. The protein-DNA complex was resolved on a 5% non-denaturing polyacrylamide gel and the gel image was obtained by the quantitative infrared fluorescent imaging system. The unlabeled *flhDC* promoter DNA acted as a competitor to verify the binding specificity. comDNA, competitive DNA fragments. (**e**) A diagram showing the Crp-cAMP binding sites upstream of *flhDC* gene. The putative Crp-cAMP binding sequences are underlined and the putative −10 and −35 promoter sequences of sigma 70 are shadowed. (**f**) The flagellin levels of wild-type, *crp* mutant and *crp*-complemented strain. Flagellin levels were examined after seeding on the swarming plates for 4 h by the SDS-PAGE. The band of flagellin is 40 kD. M, molecular weight marker. (**g**) TEM pictures of wild-type in the absence or presence of 10% glucose, *crp* mutant and the *crp*-complemented strain. Bacterial cultures were applied onto a carbon-coated grid, cells were stained with 1% PTA and TEM pictures were taken. Flagella are indicated by arrows. In (**a–c**,**f** and **g**), wt, wild-type; wt-10%glc, wild-type with 10% glucose; crp, *crp* mutant; crpc, *crp*-complemented strain. Full-length gels for (**d**) and (**f**) are shown in Supplementary Fig. S8.

In general, the flagella of bacterial pathogens assist in colonization and dissemination within the host^13^. In addition, antigenic variation through flagellin gene rearrangement enables *P*. *mirabilis* to evade host immune response during catheter-associated UTIs^13^. Because Crp-cAMP regulates the expression of flagella in *E*. *coli* and *S*. *marcescens* ^21, 22^, swimming and swarming assays were performed. The *crp* mutant exhibited almost no swimming (Supplementary Fig. S4) and swarming (Fig. 3a) motility compared with the wild-type and crpc strains. In addition, 10% glucose had an obvious inhibitory effect on swarming and swimming motility (Figs 3a and S4). We then examined the expression of *flhDC* by determining the promoter activity and mRNA level. We observed that *flhDC* expression was significantly reduced in the *crp* mutant compared with the wild-type and crpc strains (Fig. 3b,c). An electrophoretic mobility shift assay (EMSA) with purified His-tagged Crp and the infrared (IR) dye-labeled *flhDC* promoter DNA fragment was conducted to determine whether Crp-cAMP can directly bind the putative *flhDC* promoter in *P*. *mirabilis*. Polyacrylamide gel electrophoresis (PAGE) revealed a band shift after the labeled *flhDC* promoter DNA was incubated with adequate His-tagged Crp, and the unlabeled *flhDC* promoter DNA acted as a competitor in reducing the binding of the IRDye-labeled *flhDC* promoter DNA with His-Crp (Fig. 3d), in the presence of a predicted Crp binding site in the *flhDC* promoter region (Fig. 3e). No band shift was observed in a sample from the IRDye-labeled negative control DNA incubated with His-Crp (Supplementary Fig. S7). Accordingly, an analysis of flagellin expression through sodium dodecyl sulfate-PAGE revealed that the *crp* mutant produced almost no flagellin compared with the wild-type and crpc strains (Fig. 3f). Further transmission electron microscopy validated that the *crp* mutant lacked flagella (Fig. 3g). In contrast to the fewer flagella seen in the 10% glucose-treated wild-type strain (Fig. 3g), the wild-type and crpc strains had many flagella. Notably, the *crp* mutant cells failed to differentiate into elongated swarmer cells at 4 h after inoculation as opposed to the wild-type strain (Fig. 3g). Moreover, transcriptome analysis indicated that the loss of *crp* reduced the *flhDC* mRNA level (Supplementary Excel file). These findings suggest a contribution by the downregulation of flagella in the *crp* mutant to immune evasion and consequent colonization during infection.

### Loss of *P*. *mirabilis crp* increased adhesion to kidney epithelial cells and mRNA expression of Pmp fimbriae

**Figure 4:**
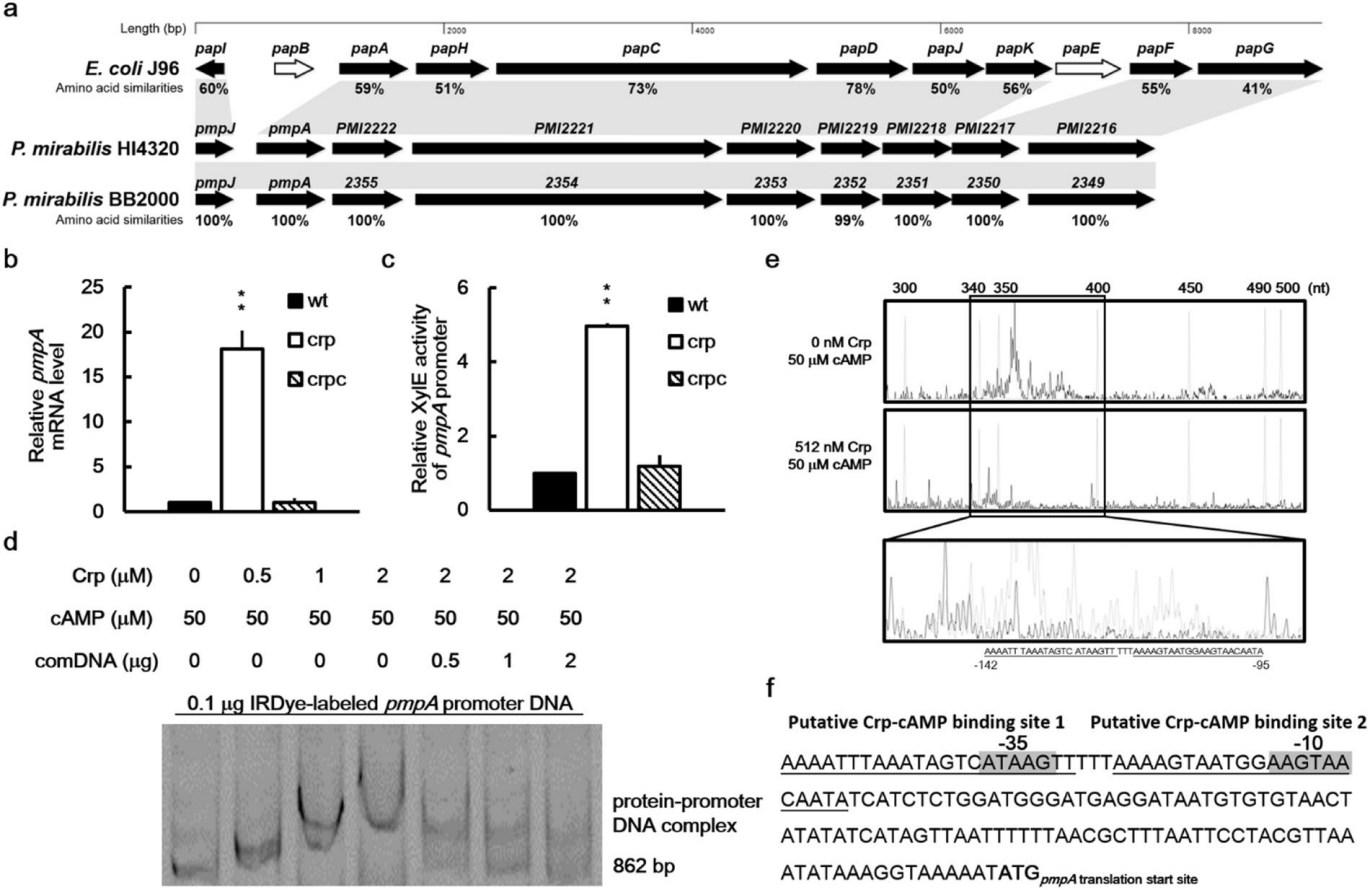
Regulation of *pmpA* expression by *P*. *mirabilis* Crp-cAMP. (**a**) The P-like fimbrial gene locus (*pmp*) similar to the well characterized *pap* locus in UPEC J96 in *P*. *mirabilis* genome. An amino acid sequence analysis of the *pmp* locus in *P*. *mirabilis* HI4320 (accession no. AM942759) and its counterparts in *E*. *coli* J96 (accession no. ALIN02000070) and *P*. *mirabilis* BB2000 (accession no. NC_022000) was performed using position-specific iterative BLAST. The nine proteins of the *pmp* locus in *P*. *mirabilis* HI4320 or BB2000 are similar to the PapI, PapA, PapH, PapC, PapD, PapJ, PapK, PapF and PapG in *E*. *coli* J96 with corresponding genes in shadows. The percent amino acid similarities between *P*. *mirabilis* HI4320 and *E*. *coli* J96 or *P*. *mirabilis* BB2000 were shown below each gene. The white arrows represent genes that are not found in either *P*. *mirabilis* HI4320 or *P*. *mirabilis* BB2000. (**b**) Loss of *crp* increased the *pmpA* mRNA level. The *pmpA* mRNA levels of the wild-type, *crp* mutant, and *crp*-complemented strain were quantified using real-time RT-PCR at 24 h after incubation. The value obtained for the wild-type was set at 1. (**c**) Loss of *crp* increased the promoter activity of *pmpA*. The activities of XylE in the *pmpA-xylE* reporter plasmid-transformed wild-type, *crp* mutant, and *crp*-complemented strain were determined using the reporter assay at 7 h after incubation. The value obtained for the wild-type was set at 1. In (**b** and **c**), the data represent the averages and standard deviations of three independent experiments. The significant difference from the wild-type was determined by Student’s *t* test (**P < 0.01). wt, wild-type; crp, *crp* mutant; crpc, *crp*-complemented strain. (**d**) The binding of *P*. *mirabilis* Crp-cAMP to *pmpA* promoter region revealed using an EMSA. The EMSA was performed as described in Fig. 3d, except that IRDye-labeled *pmpA* promoter region DNA fragments (862 bp) were incubated with 0–2 μM of the purified His-tagged Crp. (**e**) Identification of the Crp-cAMP binding site in the *pmpA* promoter region by a DNase I footprinting assay. FAM was used to label the *pmpA* promoter DNA fragment and the DNA fragment was incubated with cAMP (50 μM) with or without the recombinant Crp (512 nM) followed by DNase I treatment. The mixture was subject to electrophoresis. The fluorescence intensity of the FAM-labeled DNA fragment (ordinate) was plotted against the sequence length of the fragment. Two Crp-cAMP binding sites (underlined) located between −95 and −142 upstream of *pmpA* start codon were shown in an expanded view. (**f**) A diagram showing the Crp-cAMP binding sites upstream of *pmpA* gene. The putative Crp-cAMP binding sequences are underlined and the putative −10 and −35 promoter sequences of sigma 70 are shadowed. The full-length gel for (**d**) is shown in Supplementary Fig. S8.

Because *P*. *mirabilis* Crp activity was involved in kidney colonization in diabetic mice and the loss of *crp* affected kidney colonization in nondiabetic mice, we determined the Crp-associated virulence factors participating in mouse colonization. Adherence is a key initiating step in urinary tract colonization. Accordingly, critical components of *P*. *mirabilis* pathogenesis center on fimbriae, which mediate adherence to epithelial cells of the urinary tract as well as catheters^15, 17^, and loss of Mrp fimbriae correlates with decreased bladder colonization of mice^37^. Therefore, we first performed a cell adhesion assay by using kidney epithelial cells (A498) and bladder urothelial cells (NTUB1). We observed that the *crp* mutant exhibited an increased adhesion ability to A498 cells but a decreased adhesion ability to NTUB1 cells compared with the wild-type and crpc strains; similarly, 10% glucose increased the adhesion of the wild-type strain to A498 cells but reduced the adhesion to NTUB1 cells (Fig. 2d and e). The data suggest that Crp-regulated adhesion contributes to kidney colonization. Although a synergistic relationship might exist between type 1 and P fimbriae for colonization and subsequent UTIs, UPEC has the potential to predominantly express P fimbriae during the infection of the kidney epithelium^16, 38^. Consequently, we searched for the *pmp* locus in the *P*. *mirabilis* HI4320 genome. An analysis of the deduced amino acid sequences revealed that the locus may encode nine proteins, with 60%, 59%, 51%, 73%, 78%, 50%, 56%, 55%, and 41% similarity to UPECJ96 (accession no. ALIN02000070) *papI*, *papA*, *papH*, *papC*, *papD*, *papJ*, *papK*, *papF*, and *papG*, respectively (Fig. 4a). In view of activating *papA* expression by Crp in *E*. *coli* ^39^, we examined the effect of *crp* loss on *pmpA* expression, encoding the major subunit protein of *P*. *mirabilis* P-like fimbriae, in the wild-type strain, *crp* mutant, and crpc strain by using real-time RT-PCR and a *pmpA*-*xylE* reporter assay. The results indicate that Crp negatively regulated *pmpA* expression (Fig. 4b,c). An EMSA with a negative control (Supplementary Fig. S7) revealed a visible band shift in the sample of the labeled *pmpA* promoter DNA incubated with His-Crp and that the unlabeled *pmpA* promoter DNA can act as a competitor to reduce the binding of the IRDye-labeled *pmpA* promoter DNA with His-Crp (Fig. 4d). We further probed the binding site of Crp in the upstream region of *pmpA* using the DNase I footprinting assay. The assay revealed two Crp binding sites located between −95 and −142 upstream of *pmpA* start codon (Fig. 4e). The Crp binding sites of the *pmpA* promoter overlapped the −10 and −35 regions of the putative *pmpA* promoter (Fig. 4f), as opposed to the binding site upstream of the −10 and −35 regions of the *E*. *coli papA* promoter^39^.

**Figure 5:**
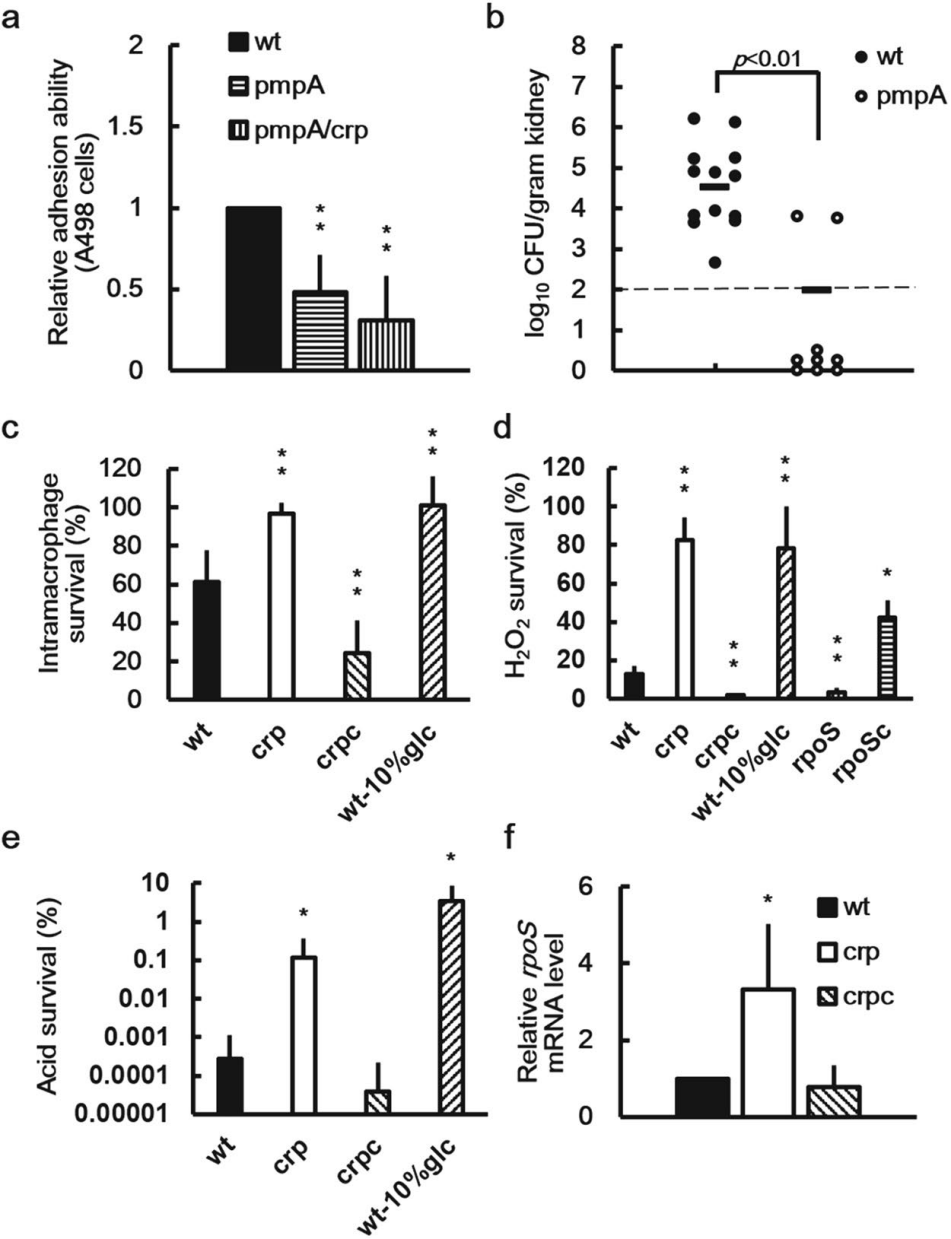
Loss of *pmpA* abolished the increased kidney adhesion of *crp* mutant and decreased kidney colonization, and *crp* mutant displayed enhanced stress tolerance and upregulation of RpoS. (**a**) Adhesion of wild-type *P*. *mirabilis*, *pmpA* mutant or *pmpA*/*crp* double mutant to A498 cells. Adhesion abilities were determined as in Fig. 2. (**b**) Mouse kidney colonization by the wild-type or *pmpA* mutant. Colonization was determined as in Fig. 2b. (**c**) Intramacrophage survival of the wild-type in the absence or presence of 10% glucose, *crp* mutant and *crp*-complemented strain. THP-1 cells were infected with bacteria for 30 min at an MOI of 10. Intramacrophage survival was determined as the percentage of viable bacteria that survived in the macrophages for 4 h versus those for 1 h after outside bacteria were killed with streptomycin. (**d**) Survival in H_2_O_2_ of wild-type in the absence or presence of 10% glucose, *crp* mutant, *crp*-complemented strain, *rpoS* mutant and *rpoS*-complemented strain. Overnight cultures were regrown and adjusted to a density of 10^8^ cells/ml before cells were exposed to 30 mM H_2_O_2_ for 20 min at 37 °C. The percent cell survival was determined by colony counting relative to the untreated control. (**e**) Survival in acid of wild-type in the absence or presence of 10% glucose, *crp* mutant and *crp*-complemented strain. Overnight bacterial cultures were regrown before cells were challenged with acid (pH 3.0) for 2 h. The percent cell survival was obtained as in (**d**). (**f**) Loss of *crp* increased the *rpoS* mRNA level. The mRNA amounts in the wild-type, *crp* mutant and *crp*-complemented strain were quantified sing real-time RT-PCR at 3 h after incubation. The value obtained for the wild-type was set at 1. In (**a**,**c**–**f**), the data represent the averages and standard deviations of three independent experiments. The significant difference from the wild-type was determined by Student’s *t* test (*P < 0.05; **P < 0.01). wt, wild-type; pmpA, *pmpA* mutant; crp, *crp* mutant; pmpA/crp, *pmpA*/*crp* double mutant; crpc, *crp*-complemented strain; wt-10%glc, wild-type with 10% glucose; rpoS, *rpoS* mutant; rpoSc, *rpoS*-complemented strain.

To verify the significance of Crp-regulated PmpA in adhesion to A498 cells, we performed a cell adhesion assay involving an isogenic *pmpA* mutant (Supplementary Fig. S3) and *pmpA/crp* double mutant. A nearly 50% reduction in adhesion was observed for the *pmpA* mutant compared with the wild-type strain (Fig. 5a), and the adhesion ability of the *pmpA/crp* double mutant was comparable to that of the *pmpA* mutant (Fig. 5a). Subsequently, we assessed whether Pmp fimbriae (PmpA) played an important role in kidney colonization in mice. Kidney colonization by the *pmpA* mutant was significantly attenuated compared with that by the wild-type strain (Fig. 5b). In conclusion, a large part of the increased adhesion to kidney cells of the *crp* mutant could be attributed to the upregulation of Pmp fimbriae. Moreover, the transcriptome analysis through RNA-seq indicates that *pmp* genes were upregulated in both the *crp* mutant and 10% glucose-treated wild-type strain (Supplementary Excel file).

### Loss of *crp* increased survival of *P*. *mirabilis* in macrophages

Successful colonization of the urinary tract requires *P*. *mirabilis* to overcome a barrage of innate host defenses, including killing by macrophages and the generation of reactive oxygen species (ROS)^13^. To determine whether Crp is involved in counteracting the innate ability of macrophages to eliminate *P*. *mirabilis*, we challenged macrophages with the wild-type strain or its derivatives, killed bacteria outside the macrophages with streptomycin, and assessed the survival of internalized bacteria. No significant difference was observed between the colony-forming units (CFU) of the wild-type strain and *crp* mutant within macrophages immediately after outside bacteria were killed with streptomycin. The relative survival of the wild-type strain was reduced to approximately 60% at 4 h versus 1 h after outside bacteria were killed with streptomycin (Fig. 5c). However, the survival of the *crp* mutant or 10% glucose-treated wild-type strain remained 100% (Fig. 5c). The crpc strain exhibited a significant reduction in relative survival compared with the wild-type strain, possibly because of the overexpression of Crp in the crpc strain (Fig. 1b).

### Loss of *crp* increased the resistance of *P*. *mirabilis* to H_2_O_2_ and acid

Because *crp* expression and Crp activity affected the intramacrophage survival of *P*. *mirabilis*, we investigated whether Crp was involved in coping with challenges within the urinary tract or macrophages such as oxidative and acid stresses during infection. The survival of the wild-type strain, *crp* mutant, and crpc strain was assessed after exposure to H_2_O_2_ or acid. The loss of *crp* significantly increased survival after exposure to 30 mM H_2_O_2_ or HCl at pH 3 (Fig. 5d,e). Furthermore, the 10% glucose-treated wild-type strain was more resistant to H_2_O_2_ and acid than the wild-type strain (Fig. 5d,e). The crpc strain was nearly as susceptible to stresses as the wild-type strain or more susceptible than the wild-type strain (Fig. 5d,e).

### Loss of *crp* increased the expression of sigma factor RpoS of *P*. *mirabilis*

Crp has been reported to regulate the expression of the general stress response regulator RpoS of *E*. *coli* ^40^. We demonstrated that loss of *rpoS* rendered *P*. *mirabilis* more sensitive to H_2_O_2_ than the wild-type strain (Fig. 5d). Previously, we found that *P*. *mirabilis* Hfq positively regulated the expression of *rpoS* mRNA^41^. In the present study, we demonstrated that Crp negatively regulated the expression of *hfq* mRNA (Fig. 1b) and Crp participated in the tolerance to H_2_O_2_ and acid (Fig. 5d,e). Hence, we investigated the effect of *crp* loss on the expression of *rpoS*. We observed that *rpoS* mRNA level was increased in the *crp* mutant compared with the wild-type and crpc strains (Fig. 5f). Accordingly, transcriptome data also revealed that the loss of *crp* increased the expression of *rpoS* (Supplementary Excel file). These results suggest that the loss of *crp* or high-glucose conditions (low Crp activity) protect *P*. *mirabilis* from stressful conditions (acid or H_2_O_2_) through the RpoS-dependent pathway.

## Discussion

The Crp regulatory system is a paradigm of bacterial gene regulation and was extensively investigated in *E*. *coli* and *Salmonella* in a prior study^42^. However, despite decades of intensive studies, new information has continued to emerge. Crp-cAMP was previously coopted to control different regulons in bacteria that occupy distinct niches^4^. In this study, we characterized *P*. *mirabilis* Crp in terms of the response to glucose, gene regulation, and virulence traits including host colonization, fimbria-mediated adhesion, stress tolerance, and motility. We provided several lines of evidence to demonstrate the Crp counterpart in uropathogenic *P*. *mirabilis*. First, *P*. *mirabilis* Crp shares a high sequence identity with its homologue in other bacteria. Second, *P*. *mirabilis* Crp activity was associated with the glucose level, and the loss of *crp* caused defects in growth and metabolism. Third, *P*. *mirabilis* Crp-cAMP regulated the expression of *ptsG*, *cyaA*, and *hfq* similarly to how it did in *E*. *coli*. In addition, *P*. *mirabilis* Hfq positively regulated the expression of *crp*, as in *Yersinia* ^7^. In the normal LB culture condition, the CyaA of *P*. *mirabilis* can exist in an active form, catalyzing the conversion of ATP to cAMP. Once the Crp-cAMP complex was formed, CyaA positively regulated the expression of *ptsG*, but negatively regulated the expression of *hfq* and *cyaA*, possibly by binding to the respective promoter region. In *E*. *coli*, extracellular glucose is taken up by the PtsG transporter. High glucose levels inhibit CyaA activity, resulting in decreased cAMP levels and the repression of PtsG, thus forming the feedback regulation of glucose uptake^10^. Our data suggest that the feedback regulation of PtsG by Crp-cAMP in glucose uptake exists in *P*. *mirabilis*. Many studies have demonstrated that *crp* expression must be appropriately controlled to ensure optimal cell physiology. Environmental alterations during infection could serve as signals to activate the TCS or trigger the expression of regulatory small RNAs (sRNAs) that influence Crp expression in *P*. *mirabilis*. For example, *Y*. *pestis crp* is regulated by the PhoPQ TCS^11^. Our data suggest that Hfq-dependent sRNAs are likely to posttranscriptionally stimulate Crp synthesis, thereby affecting the expression of the Crp regulons such as *pmp* during infection. Additional studies are required to further delineate the sRNAs that contribute to the regulation of Crp and the host signals detected by *P*. *mirabilis* to influence the Crp regulon.

Crp is a regulator of not only genes involved in the catabolism of sugars^1, 3^ but also a multitude of genes associated with stress tolerance and virulence^2, 3, 43, 44^. The involvement of Crp, a bacterial factor, in kidney rather than bladder colonization in diabetic mice, as well as increased colonization in nondiabetic mice in the kidney but not in the bladder by the *crp* mutant, as revealed in this study (Fig. 2b,c), indicates the complexity of gene regulation by Crp in different niches. UPEC Crp-cAMP was reported to be responsible for effective bladder colonization in a mouse infection model, probably due to the regulatory effects of Crp-cAMP on factors required for resistance to stresses generated in the niche^36^. In this study, bladder colonization in nondiabetic mice by the *P*. *mirabilis crp* mutant was not significantly attenuated (Fig. 2c); however, Mrp fimbriae (a factor for bladder colonization by *P*. *mirabilis*) were downregulated in the *crp* mutant (Supplementary Fig. S2). Other factors, such as resistance to H_2_O_2_ and killing by macrophages, may counteract the effect of Mrp fimbriae on bladder colonization by the *crp* mutant in nondiabetic mice. Notably, the *P*. *mirabilis crp* mutant was more successful in colonizing the kidneys of nondiabetic mice than the wild-type strain (Fig. 2b). Yet the effect of the loss of *crp* on kidney colonization remains to be addressed. Our data suggest that Crp-regulated P-like fimbriae (Pmp), survival in macrophages, stress tolerance, and immune evasion due to flagellum deficiency are involved in this process. Our study was the first to demonstrate the inhibition of *P*. *mirabilis* Crp activity in the presence of high glucose levels (Fig. 1a) and its significantly lower level in diabetic mice than in nondiabetic mice (Fig. 2a). Because the urine glucose concentration of normal adults ranges from 0.002% to 0.02% (2–20 mg/dL) and that in people with diabetes is higher than 3.14% (3140 mg/dL) (http://www.urinemetabolome.ca/), Crp inactivation might be biologically significant, leading to increased kidney colonization and subsequent infection in patients with diabetes.

P fimbriae, as a prominent feature of UPEC, mediate its adherence and persistence in mammalian kidneys^16, 45^. P fimbria adhesin (PapG) is known to bind to the globoseries of glycolipids abundantly expressed on the surface of kidney cells^46^. The similarity of *P*. *mirabilis* putative adhesin encoded by PMI2216 (one gene of the *pmp* operon) to PapG indicates that *P*. *mirabilis* P-like fimbriae (Pmp) might mediate adhesion to kidney cells, as in UPEC. Hence, we conducted the first investigation of the influence of *P*. *mirabilis* P-like fimbriae on kidney adhesion and colonization and the regulation of P-like fimbriae by Crp. Several lines of evidence in this study support that the upregulation of P-like fimbriae in the *crp* mutant accounts for a large part of the enhanced kidney colonization. First, the *crp* mutant exhibited increased adhesion to kidney cells (Fig. 2d), and the *pmpA* mutant exhibited a reduced kidney adhesion ability comparable to that of the *pmpA/crp* double mutant (Fig. 5a). Second, the loss of *crp* upregulated *pmpA* expression (Fig. 4b,c). Furthermore, EMSA (Fig. 4d) and DNase I footprinting (Fig. 4e) validated that Crp can bind to the Crp box located in the promoter region of *pmpA* (Fig. 4f). Third, colonization of the kidney by the *pmpA* mutant was significantly attenuated (Fig. 5b). The upregulation of *pmpA* (P-like fimbriae) expression in the *crp* mutant also implies that the increased kidney colonization in diabetic mice by wild-type *P*. *mirabilis* compared with nondiabetic mice might have resulted from the increased expression of P-like fimbriae in the diabetic condition (high glucose) with reduced Crp activity.

The UPEC Crp-cAMP complex plays a positive regulatory role in *pap* transcription^47^. Unlike UPEC, the expression of *P*. *mirabilis pmpA* was under the negative control of Crp-cAMP. Because Crp-cAMP can act as an activator or a repressor^1–3^, the mode of transcriptional regulation by Crp-cAMP could correlate with its DNA binding site relative to the promoter of the target gene^48^. The discrepancy of P fimbria regulation by Crp between *P*. *mirabilis* and *E*. *coli* may be because of the overlapping of the Crp-cAMP binding site with the −35 and −10 regions of the *pmpA* promoter in *P*. *mirabilis* but not in UPEC^49^. The *E*. *coli pap* promoter is highly regulated by numerous factors, including PapI, Lrp, Cpx, and Crp^47, 49, 50^. Recently, RcsB, a two-component response regulator, was reported to increase *pmpA* expression in *P*. *mirabilis* (www.ncbi.nlm.nih.gov/geo/; accession number: GSE76341). Investigation of the molecular mechanisms of *pmp* expression in *P*. *mirabilis* is therefore warranted.

Bacterial flagellin is recognized by Toll-like receptor 5, resulting in inflammatory cytokine production, immune cell recruitment to the infectious site, and elimination of the invading bacteria^51^. In the urinary tract, the immune mechanisms of the host favor the removal of bacteria expressing immunogenic surface structures such as flagella^13^. In this regard, we observed that the *P*. *mirabilis crp* mutant produced almost no flagella (Fig. 3g). This finding implies that Crp participates in the regulation of expression of the surface flagellin protein to alter the immunogenicity of the bacterium for preventing clearance by the host. Similarly, Crp-cAMP regulates flagellin expression in *E*. *coli* and *S*. *marcescens* ^21, 22^. Although flagella are believed to contribute to the virulence of uropathogenic *P*. *mirabilis* by allowing movement from the catheter to the bladder and the kidney in ascending UTIs, *P*. *mirabilis* mutants lacking flagella were demonstrated to cause ascending UTIs in mice^52^. Additional studies are required to determine the movement of the *P*. *mirabilis crp* mutant lacking flagella to the kidneys.

Although *P*. *mirabilis* is not an intracellular pathogen, intramacrophage survival during early infection was proposed to be an important fitness index^53^. The increased number of surviving *crp* mutant cells inside macrophages and the increased ability of the *crp* mutant to withstand H_2_O_2_ and acid (Fig. 5c,d and e) indicate that the loss of *crp* can improve the fitness of *P*. *mirabilis* during infection. RpoS, a bacterial stationary phase sigma factor, regulates the expression of various genes to enable bacteria to overcome multiple environmental stresses, including ROSs such as H_2_O_2_^54, 55^. In addition, *rpoS* transcription was demonstrated to be regulated by Crp-cAMP^56^, and Crp could inhibit RpoS expression through Spf, an Hfq-dependent sRNA that facilitates acid resistance by activating RpoS expression in *E*. *coli* ^40^. Our unpublished data also revealed that *P*. *mirabilis* Crp negatively regulated Spf expression, and the loss of *hfq* reduced the Spf RNA level. The previous finding that *P*. *mirabilis* Hfq positively regulated RpoS expression^41^, along with the present study’s findings of Crp-mediated negative regulation of *hfq* mRNA expression, increased tolerance of the *crp* mutant to H_2_O_2_ and upregulation of *rpoS* mRNA in the *crp* mutant (Fig. 5f), and decreased H_2_O_2_ resistance in the *rpoS* mutant (Fig. 5d), suggest that the upregulation of the RpoS pathway through Hfq due to the loss of *crp* strengthens the resistance of *P*. *mirabilis* to stressful environments.

This is the first study to report that *P*. *mirabilis* Crp can regulate multiple virulence traits, including colonization of mice, production of flagella, fimbria-mediated adhesion, survival in macrophages, and stress tolerance in response to glucose. We demonstrated that the upregulation of P-like fimbriae, enhanced stress tolerance, and host immune evasion due to flagellum deficiency in the *crp* mutant facilitated kidney colonization. Hence, the reduced Crp activity in a high-glucose environment in diabetic mice may contribute to the increased kidney colonization by *P*. *mirabilis*. Moreover, this study shows that *P*. *mirabilis* is capable of optimizing metabolic and virulence gene expression through the Crp regulon to link catabolite repression with multiple regulatory pathways involving Hfq and sRNAs. Understanding the regulatory network that governs the expression of virulence or colonization factors might facilitate the development of novel strategies for treating bacterial infections. Therefore, we suggest that P-like fimbriae can serve as a target for the design of antibacterial strategies. For this purpose, a recombinant strain *E*. *coli* 83972::*lgtCE*, a nonpathogenic *E*. *coli* strain exhibiting a P fimbria receptor mimic on its surface^46^, may act as a prophylactic agent for preventing UTIs caused by *P*. *mirabilis*.

## Methods

### Bacterial strains, plasmids, reagents and growth conditions

The bacterial strains and plasmids used in this study are listed in Supplementary Table S1. Construction of *P*. *mirabilis* mutants and *crp*-complemented strain were described in the Supplemental Material. All chemicals were obtained from the Sigma-Aldrich unless otherwise indicated, and primer sequences are listed in Supplementary Table S2. Bacteria were routinely cultured in Luria-Bertani (LB) broth at 37 °C. The LSW^−^ agar was used to prevent the phenotypic expression of swarming motility for selecting mutant clones and determining colony forming units (CFU). Ampicillin (100 μg/ml), chloramphenicol (20 μg/ml), gentamicin (50 μg/ml), kanamycin (100 μg/ml), tetracycline (20 μg/ml), or glucose (2 or 10% (2000 or 10000 mg/dl)) was added to the medium as needed.

### Real-time reverse transcription PCR (RT-PCR)

To study the effect of *crp* loss on the expression of *ptsG*, *hfq*, *cyaA*, *crp*, *pmpA*, *flhDC*, *rpoS* and *mrpA* mRNAs, overnight LB cultures of the wild-type, *crp* mutant and the crpc strain were diluted 100-fold in the same medium and incubated for 3 h (*rpoS*), 7 h (*ptsG*, *hfq*, *cyaA*, *crp*, *flhDC*), or 24 h (*pmpA*, *mrpA*) at 37 °C. Total RNA was extracted, and real-time RT-PCR was carried out as described previously^57^ to monitor the mRNA expression normalized against the *gyrB* mRNA.

### GFPuv and XylE reporter assays

Because the *lac* promoter is positively regulated by Crp-cAMP, a p*lac*-*gfpuv* reporter plasmid was constructed to assess the effect of glucose on the activity of Crp-cAMP in *P*. *mirabilis* by measuring GFP fluorescence intensity. *gfpuv* gene fragment was obtained by PCR from pGFPuv (Clontech) and cloned into the TA cloning vector, pGEM-T Easy, to produce pGgfpuv, a p*lac*-*gfpuv* reporter plasmid; *gfpuv* gene is thus driven by the *lac* promoter in the plasmid. The wild-type *P*. *mirabilis* transformed with pGgfpuv was grown overnight in LB broth containing ampicillin. The cultures were diluted 100-fold in the same medium with 0%, 2%, or 10% glucose and incubated for 3, 5, 7, 9, 11 and 24 h at 37 °C. One milliliter of the cell suspension at each time point was centrifuged and the pellet was re-suspended in sterile phosphate-buffered saline (PBS) (1 ml) for fluorescence intensity determination (excitation at 395 nm and emission at 507 nm) normalized against the OD_600_ value.

For the XylE reporter assay, the promoter region of the *crp*, *pmpA* or *flhDC* was amplified using SphI and PstI-included primer pairs (Supplementary Table S2) and cloned into the pGEM-T Easy. These promoter-containing plasmids were cut by SphI and PstI, and the promoter-containing fragment was cloned, respectively to the *xylE*-containing pACYC184 to construct the different reporter plasmids. The wild-type, *crp* mutant, and crpc strain were transformed with respective reporter plasmids and grown overnight in LB broth containing chloramphenicol. The cultures were diluted 100-fold in the same medium and incubated for 7 h at 37 °C. The XylE activity was measured as described previously^58^.

### Animal experiments

Induction of diabetes in ICR mice was performed as described previously^59^ with modifications. Six-week-old female mice were treated with two consecutive daily intravenous (i.v. 125 mg/kg body weight) injections of streptozotocin (STZ, Sigma-Aldrich) freshly dissolved in sodium citrate buffer (50 mM sodium citrate, pH 4.5) to induce β-cell death of the pancreatic islet. Control mice were treated with the same amount (50 μl) of citrate buffer without STZ. On day one, all food except water was removed from cages at 4 h prior to STZ treatment. After injection, all mice were fed with a normal diet. Blood and urine glucose levels were measured daily after three days of the second STZ injection. Mice with two consecutive blood glucose levels ≥250 mg/dl were considered diabetic. All STZ-injected mice became diabetic within 1 week after the second STZ injection.

The mouse model of UTIs was used as described previously^60^. Six-week-old female ICR mice were injected transurethrally with overnight cultures of bacteria at a dose of 10^7^ CFU per mouse. On day 3 after injection, mice were sacrificed and bladder and kidney samples were collected to determine the viable bacterial count. All animal experiments were performed in strict accordance to the recommendations in the Guide for the Care and Use of Laboratory Animals of the National Laboratory Animal Center (Taiwan), and the protocol was approved by the Institutional Animal Care and Use Committee of National Taiwan University College of Medicine.

### Cell adhesion assay

Human A498 kidney epithelial cells and human NTUB1 bladder urothelial cells^57^ were grown on 12-well cell culture plates (Corning) in 10% fetal bovine serum-containing advanced Minimum Essential Medium (MEM, Gibco) and RPMI-1640 medium (Gibco), respectively, to confluent monolayers before being washed with PBS. Overnight bacterial cultures were centrifuged and the pellet was re-suspended in MEM or RPMI-1640 before applying to each well at an MOI of 10 (10^7^ CFU/well). Bacteria were brought into contact with epithelial cells by centrifugation, followed by incubation for 1 h at 37 °C. Monolayers of cells were lysed with 1% Triton X-100 to determine bacterial CFU after being washed with PBS. The cell adhesion ability was the percentage of viable bacteria that adhered to the A498 cells or NTUB1 cells versus the total inoculum, and the relative adhesion ability to the wild-type was expressed.

### Swarming assay

The swarming assay was performed as described previously on LB agar (1.5%, w/v) plates^41^ with or without 10% glucose. The swarming migration distance was monitored by following swarm fronts of the bacterial cells and recording progress at 60-min intervals.

### Measurement of the flagellin level

Flagellin levels were determined as described previously^41^. The bacterial suspension washed from the LB agar plate with PBS (10 ml, OD_600nm_ = 0.6) was used to prepare flagellin by vigorous vortexing and subsequent centrifugation. Flagellin in the supernatant was precipitated with trichloroacetic acid (5%, w/v), separated on 12% SDS-PAGE and stained with Coomassie Brilliant Blue R (Sigma-Aldrich B7920).

### Transmission electron microscopy (TEM)

TEM was performed as described previously by using 1% phosphotungstic acid (PTA)-stained bacteria on a carbon-coated grid and TEM pictures were obtained with a Hitachi H-7100 electron microscope^41^.

### Expression and purification of the His-tagged recombinant Crp

*crp* was cloned into the plasmid pET32a(+) (Novagen) to generate the pET32a-crp plasmid. Overexpression and purification of His-tagged Crp were performed as described previously^57^. An overnight culture of *E*. *coli* BL21 harboring pET32a-crp was diluted and grown at 37 °C with vigorous shaking to an optical density at 600 nm of 0.5 to 0.6 followed by addition of isopropyl-β-D- thiogalactopyranoside (IPTG) (0.1 mM) to induce Crp expression. The His-tagged Crp was purified by using a Ni^2+^-nitrilotriacetic acid column (Invitrogen) and its purity was confirmed by SDS-PAGE (Supplementary Fig. S5).

### Electrophoretic mobility shift assay (EMSA)

An EMSA using purified His-tagged Crp and the IRDye-labeled *flhDC* or *pmpA* promoter DNA in the presence of cAMP was performed. The unlabeled promoter DNA fragment acted as a competitor to verify the binding specificity. The *flhDC* and *pmpA* promoter sequence were PCR- amplified by using the primer pairs flhDC-reF/flhDC-reR and pmp-fpF/pmp-koR, respectively (Supplementary Table S2). The PCR product was cloned into pGEM-T Easy (Promega) to generate pGflhDCp or pGpmpAp. The IRDye-labeled *flhDC* and *pmpA* promoter fragments were amplified from the pGflhDCp and pGpmpAp, respectively, using the IRDye® 700-labeled M13F/M13R primers (Supplementary Table S2, Integrated DNA Technologies). The amplified product (0.1 μg) was incubated with His-Crp (0–2 or 0–0.5 μM) and cAMP (50 μM) in a 10-μl binding buffer (150 mM KCl, 10 mM MgCl_2_, 30 mM Tris-HCl, 50 μM cAMP, pH 8.0). After incubation for 30 min at room temperature, the reaction mixtures were loaded onto a 5% non-denaturing polyacrylamide gel buffered with Tris-acetate-EDTA containing 50 μM cAMP, and then the gel was electrophoresed at 100 V for 1.5 to 2 h. The gel image was obtained by the quantitative infrared fluorescent imaging system (Odyssey, LI-COR). The unlabeled *flhDC* and *pmpA* promoter DNA fragments (0.5, 1, 2 μg) amplified from pGflhDCp and pGpmpAp, respectively, using unlabeled M13 primers, acted as a competitor. The DNA fragment amplified from the pGEM®-T Easy without the promoter sequence by the same primer pair was used as a negative control (Supplementary Fig. S7).

### DNase I footprinting

A DNase I footprinting assay was modified from the protocol described by Yindeeyoungyeon and Schell^61^. The 597-bp fragment from nucleotide position −415 to 182 of *pmpA* start codon was PCR-amplified by using the primer pair pmp-fpF/pmp-koR and cloned into pGEM-T Easy to generate the plasmid pGpmp-fp. The 6-carboxyfluorescein (FAM)-labeled DNA fragment was generated by using PCR with the 5′ FAM-labeled T7 primer (Genomics, Taiwan) and the unlabeled SP6 primer. The FAM-labeled DNA fragment (0.2 μg) was incubated with Crp (512 nM) in a 150-μl solution containing 10 mM Tris-HCl (pH 7.5), 0.1 mM EDTA, 5.5 mM MgCl_2_, 20 μg/ml of poly(dI-dC), 50 μM cAMP and 0.2 mg/ml of BSA for 30 min at room temperature. After addition of freshly prepared DNase I solution (150 μl) containing 10^−4^ U/μl DNase I (Epicentre, USA), 20 mM Tris-HCl (pH 7.5), 5 mM MgCl_2_, and 1 mM CaCl_2_, the mixture was further incubated at 26 °C for 5 min, followed by incubation at 37 °C for 30 min in a stop solution (300 μl) containing 0.2 M NaCl, 40 mM EDTA, 1% SDS, and 125 μg/ml of tRNA to stop DNase I digestion. The DNA sample was extracted with a phenol:chloroform:isoamyl alcohol solution (25:24:1), precipitated with absolute ethanol, washed with 70% ethanol, and dissolved in 10 μl deionized water. A portion of the DNA sample (0.5 μl) combined with the LIZ-500 molecular size standard (Applied Biosystems) (0.5 μl) and deionized formamide (9 μl) was denatured at 95 °C for 5 min and quickly chilled on ice. Electrophoresis by using a 3730xl capillary DNA analyzer (Applied Biosystems) and an analysis by using Peak Scanner Software, version 1.0 (Applied Biosystems) were performed according to the instructions supplied by the manufacturer.

### Macrophage infection assay

The assay was performed as described previously^60^ with some modifications. Briefly, overnight bacterial cultures were applied to a 12-well plate containing differentiated THP-1 cells at an MOI of 10 (10^6^ CFU/well). After infection for 30 min, bacteria outside THP-1 cells were killed with streptomycin (250 μg/ml). The 12-well plate was incubated further in the medium containing streptomycin (32 μg/ml o) for 1 and 4 h. At each time point, cells in wells were lysed by using 1% Triton X-100 to determine CFU of intracellular bacterial cells. Intramacrophage survival was the percentage of viable bacteria that survived in the macrophages for 4 h versus those for 1 h.

### Stress tolerance assays

Acid resistance assay was performed as described by Wu *et al*. with some modifications^40^. Overnight cultures were diluted 500-fold in fresh LB broth and incubated at 37 °C with shaking at 250 rpm for 4 h. After adjusting the LB broth with HCl to pH 3.0, bacterial cells were treated statically with acid for 2 h. Cells were washed with PBS (pH 7.2) and plated on LSW^−^ plates to determine the CFU. H_2_O_2_ survival test was performed as described previously^41^, except that 30 mM H_2_O_2_ was used. The percent cell survival of acid or H_2_O_2_ was calculated as 100× (CFU of treated cells/CFU of untreated cells).

## Electronic supplementary material

supplementary dataset 1

supplementary information

## References

[1] Shimada T, Fujita N, Yamamoto K, Ishihama A (2011). Novel roles of cAMP receptor protein (CRP) in regulation of transport and metabolism of carbon sources. PLoS One.

[2] Gosset G, Zhang Z, Nayyar S, Cuevas WA, Saier MH (2004). Transcriptome analysis of Crp-dependent catabolite control of gene expression in *Escherichia coli*. J. Bacteriol..

[3] Zheng D, Constantinidou C, Hobman JL, Minchin SD (2004). Identification of the CRP regulon using *in vitro* and *in vivo* transcriptional profiling. Nucleic Acids Res..

[4] Green J (2014). Cyclic-AMP and bacterial cyclic-AMP receptor proteins revisited: adaptation for different ecological niches. Curr. Opin. Microbiol..

[5] Heroven AK, Dersch P (2014). Coregulation of host-adapted metabolism and virulence by pathogenic *yersiniae*. Front. Cell Infect. Microbiol..

[6] Muller CM (2009). Type 1 fimbriae, a colonization factor of uropathogenic *Escherichia coli*, are controlled by the metabolic sensor CRP-cAMP. PLoS Pathog..

[7] Lathem WW (2014). Posttranscriptional regulation of the *Yersinia pestis* cyclic AMP receptor protein Crp and impact on virulence. MBio.

[8] Kolb A, Busby S, Buc H, Garges S, Adhya S (1993). Transcriptional regulation by cAMP and its receptor protein. Annu. Rev. Biochem..

[9] Hanamura A, Aiba H (1991). Molecular mechanism of negative autoregulation of *Escherichia coli crp* gene. Nucleic Acids Res..

[10] Shimizu K (2013). Metabolic regulation of a bacterial cell system with emphasis on *Escherichia coli* metabolism. ISRN Biochem..

[11] Zhang Y (2013). Autoregulation of PhoP/PhoQ and positive regulation of the cyclic AMP receptor protein-cyclic AMP complex by PhoP in *Yersinia pestis*. J. Bacteriol..

[12] Armbruster CE, Mobley HL (2012). Merging mythology and morphology: the multifaceted lifestyle of *Proteus mirabilis*. Nat. Rev. Microbiol..

[13] Jacobsen SM, Stickler DJ, Mobley HL, Shirtliff ME (2008). Complicated catheter-associated urinary tract infections due to *Escherichia coli* and *Proteus mirabilis*. Clin. Microbiol. Rev..

[14] Nielubowicz GR, Mobley HL (2010). Host-pathogen interactions in urinary tract infection. Nat. Rev. Urol..

[15] Jansen AM, Lockatell V, Johnson DE, Mobley HL (2004). Mannose-resistant *Proteus*-like fimbriae are produced by most *Proteus mirabilis* strains infecting the urinary tract, dictate the *in vivo* localization of bacteria, and contribute to biofilm formation. Infect. Immun..

[16] Lane MC, Mobley HL (2007). Role of P-fimbrial-mediated adherence in pyelonephritis and persistence of uropathogenic *Escherichia coli* (UPEC) in the mammalian kidney. Kidney Int..

[17] Kuan L, Schaffer JN, Zouzias CD, Pearson MM (2014). Characterization of 17 chaperone-usher fimbriae encoded by *Proteus mirabilis* reveals strong conservation. J. Med. Microbiol..

[18] Armbruster CE, Hodges SA, Mobley HL (2013). Initiation of swarming motility by *Proteus mirabilis* occurs in response to specific cues present in urine and requires excess L-glutamine. J. Bacteriol..

[19] Alteri CJ, Himpsl SD, Engstrom MD, Mobley HL (2012). Anaerobic respiration using a complete oxidative TCA cycle drives multicellular swarming in *Proteus mirabilis*. MBio.

[20] Nam TW, Park YH, Jeong HJ, Ryu S, Seok YJ (2005). Glucose repression of the *Escherichia coli sdhCDAB* operon, revisited: regulation by the CRP* cAMP complex. Nucleic Acids Res..

[21] Stella NA, Kalivoda EJ, O’Dee DM, Nau GJ, Shanks RM (2008). Catabolite repression control of flagellum production by *Serratia marcescens*. Res. Microbiol..

[22] Soutourina O (1999). Multiple control of flagellum biosynthesis in *Escherichia coli*: role of H-NS protein and the cyclic AMP-catabolite activator protein complex in transcription of the *flhDC* master operon. J. Bacteriol..

[23] Stapleton A (2002). Urinary tract infections in patients with diabetes. Am. J. Med..

[24] Nitzan O, Elias M, Chazan B, Saliba W (2015). Urinary tract infections in patients with type 2 diabetes mellitus: review of prevalence, diagnosis, and management. Diabetes Metab. Syndr. Obes..

[25] Nkume FA, Chongsi ME, Mbuntum JF, Tanjeko AT, Kwenti TE (2014). Glycemic control and urinary tract infection in diabetes mellitus: a cross sectional study. Res. Rev.: J. Med. Health Sci..

[26] Burekovic A, Dizdarevic-Bostandzic A, Godinjak A (2014). Poorly regulated blood glucose in diabetic patients-predictor of acute infections. Med. Arch..

[27] Shkurti S (2015). Prevalence of urinary tract infection among patients with diabetes melitus in Tirana district. Mediterr. J. Med. Sci..

[28] Chiţă T (2013). Prevalence of urinary tract infections in diabetic patients. Rom. J. Diabetes. Nutr. Metab. Dis..

[29] Kimata K, Takahashi H, Inada T, Postma P, Aiba H (1997). cAMP receptor protein-cAMP plays a crucial role in glucose-lactose diauxie by activating the major glucose transporter gene in *Escherichia coli*. Proc. Natl. Acad. Sci. USA.

[30] Qu S (2013). Cyclic AMP receptor protein is a repressor of adenylyl cyclase gene *cyaA* in *Yersinia pestis*. Can. J. Microbiol..

[31] Lin HH (2011). Negative effect of glucose on *ompA* mRNA stability: a potential role of cyclic AMP in the repression of *hfq* in *Escherichia coli*. J. Bacteriol..

[32] Aiba H (1985). Transcription of the *Escherichia coli* adenylate cyclase gene is negatively regulated by cAMP-cAMP receptor protein. J. Biol. Chem..

[33] Bott M (1997). Anaerobic citrate metabolism and its regulation in enterobacteria. Arch. Microbiol..

[34] Wright JM, Satishchandran C, Boyle SM (1986). Transcription of the *speC* (ornithine decarboxylase) gene of *Escherichia coli* is repressed by cyclic AMP and its receptor protein. Gene.

[35] Wu CJ, Huang YW, Lin YT, Ning HC, Yang TC (2016). Inactivation of SmeSyRy two-component regulatory system inversely regulates the expression of SmeYZ and SmeDEF efflux pumps in *Stenotrophomonas maltophilia*. PLoS One.

[36] Donovan GT, Norton JP, Bower JM, Mulvey MA (2013). Adenylate cyclase and the cyclic AMP receptor protein modulate stress resistance and virulence capacity of uropathogenic *Escherichia coli*. Infect. Immun..

[37] Bahrani FK (1994). Construction of an MR/P fimbrial mutant of *Proteus mirabilis*: role in virulence in a mouse model of ascending urinary tract infection. Infect. Immun..

[38] Melican K (2011). Uropathogenic *Escherichia coli* P and type 1 fimbriae act in synergy in a living host to facilitate renal colonization leading to nephron obstruction. PLoS Pathog..

[39] Goransson M, Forsman P, Nilsson P, Uhlin BE (1989). Upstream activating sequences that are shared by two divergently transcribed operons mediate cAMP-CRP regulation of pilus-adhesin in *Escherichia coli*. Mol. Microbiol..

[40] Wu J, Li Y, Cai Z, Jin Y (2014). Pyruvate-associated acid resistance in bacteria. Appl. Environ. Microbiol..

[41] Wang MC, Chien HF, Tsai YL, Liu MC, Liaw SJ (2014). The RNA chaperone Hfq is involved in stress tolerance and virulence in uropathogenic *Proteus mirabilis*. PLoS One.

[42] Saier, M. H., Ramseier, T. M. & Reizer, J. Regulation of carbon utilization. *in Escherichia coli and Salmonella Typhimurium: cellular and molecular biology* (eds Neidhardt, F. C., *et al*.) American Society for Microbiology, Washington, DC. **1**, 1325–1343 (1996).

[43] Chen ZW (2010). Mutations in the *Salmonella enterica* serovar Choleraesuis cAMP-receptor protein gene lead to functional defects in the SPI-1 type III secretion system. Vet. Res..

[44] Kalivoda EJ, Stella NA, O’Dee DM, Nau GJ, Shanks RM (2008). The cyclic AMP-dependent catabolite repression system of *Serratia marcescens* mediates biofilm formation through regulation of type 1 fimbriae. Appl. Environ. Microbiol..

[45] Khandige S, Kronborg T, Uhlin BE, Moller-Jensen J (2015). sRNA-mediated regulation of P-fimbriae phase variation in uropathogenic *Escherichia coli*. PLoS Pathog..

[46] Watts RE (2012). *Escherichia coli* 83972 expressing a P fimbriae oligosaccharide receptor mimic impairs adhesion of uropathogenic *E*. *coli*. J. Infect. Dis..

[47] Forsman K, Sonden B, Goransson M, Uhlin BE (1992). Antirepression function in *Escherichia coli* for the cAMP-cAMP receptor protein transcriptional activator. Proc. Natl. Acad. Sci. USA.

[48] Ishihama A (1993). Protein-protein communication within the transcription apparatus. J. Bacteriol..

[49] Hung DL, Raivio TL, Jones CH, Silhavy TJ, Hultgren SJ (2001). Cpx signaling pathway monitors biogenesis and affects assembly and expression of P pili. EMBO J..

[50] Spurbeck RR, Alteri CJ, Himpsl SD, Mobley HL (2013). The multifunctional protein YdiV represses P fimbria-mediated adherence in uropathogenic. Escherichia coli. J. Bacteriol..

[51] Steiner TS (2007). How flagellin and toll-like receptor 5 contribute to enteric infection. Infect. Immun..

[52] Legnani-Fajardo C, Zunino P, Piccini C, Allen A, Maskell D (1996). Defined mutants of *Proteus mirabilis* lacking flagella cause ascending urinary tract infection in mice. Microb. Pathog..

[53] Hamrick TS, Havell EA, Horton JR, Orndorff PE (2000). Host and bacterial factors involved in the innate ability of mouse macrophages to eliminate internalized unopsonized *Escherichia coli*. Infect. Immun..

[54] Dong T, Schellhorn HE (2010). Role of RpoS in virulence of pathogens. Infect. Immun..

[55] Battesti A, Majdalani N, Gottesman S (2011). The RpoS-mediated general stress response in *Escherichia coli*. Annu. Rev. Microbiol..

[56] Lange R, Hengge-Aronis R (1994). The cellular concentration of the sigma S subunit of RNA polymerase in *Escherichia coli* is controlled at the levels of transcription, translation, and protein stability. Genes Dev..

[57] Jiang SS (2010). *Proteus mirabilis pmrI*, an RppA-regulated gene necessary for polymyxin B resistance, biofilm formation, and urothelial cell invasion. Antimicrob. Agents Chemother..

[58] Jiang SS (2010). Characterization of UDP-glucose dehydrogenase and UDP-glucose pyrophosphorylase mutants of *Proteus mirabilis*: defectiveness in polymyxin B resistance, swarming, and virulence. Antimicrob. Agents Chemother..

[59] Wu JH, Tsai CG (2005). Infectivity of hepatic strain *Klebsiella pneumoniae* in diabetic mice. Exp. Biol. Med. (Maywood).

[60] Liu MC, Kuo KT, Chien HF, Tsai YL, Liaw SJ (2015). New aspects of RpoE in uropathogenic *Proteus mirabilis*. Infect. Immun..

[61] Yindeeyoungyeon W, Schell MA (2000). Footprinting with an automated capillary DNA sequencer. Biotechniques.

