# Supplementary material for: cAMP receptor protein regulates mouse colonization, motility, fimbria-mediated adhesion, and stress tolerance in uropathogenic Proteus mirabilis
Source: Sci Rep. 2017 Aug 4;7:7282. doi: 10.1038/s41598-017-07304-7 (PMC5544767; doi:10.1038/s41598-017-07304-7)
Supplement: Supplementary file 2 — supplementary information [file 41598_2017_7304_MOESM2_ESM.pdf]

cAMP receptor protein regulates mouse colonization, motility, fimbria-mediated adhesion, and stress tolerance in uropathogenic *Proteus mirabilis*

Yi-Lin Tsai, Hsiung-Fei Chien, Kuo-Tong Huang, Wen-Yuan Lin & Shwu-Jen Liaw

Supplementary Information

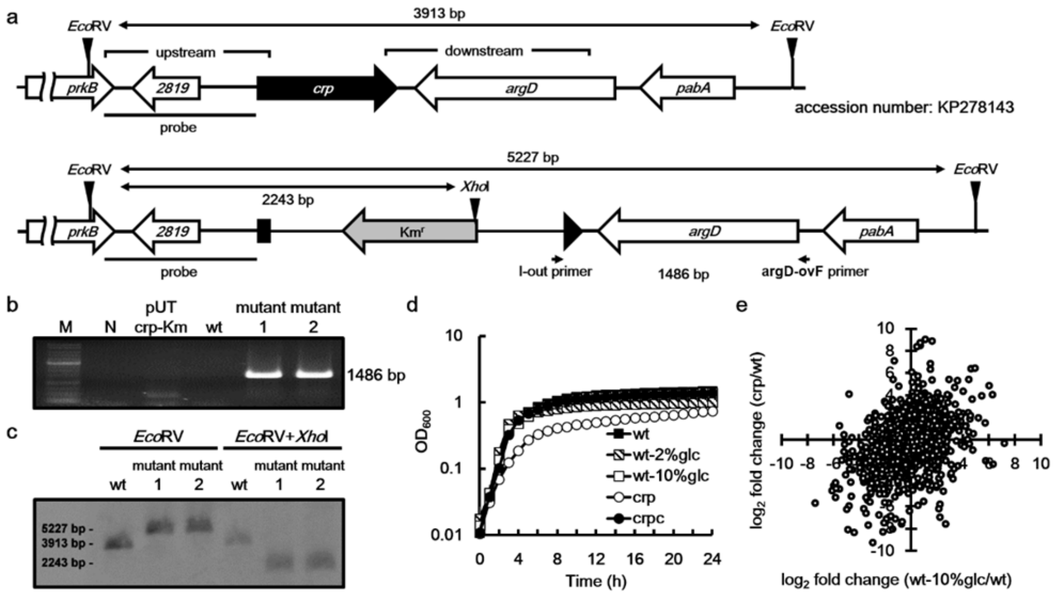

Supplementary Figure S1. (**a**)(**b**)(**c**) Construction and verification of *P. mirabilis crp* mutant. (**a**) Insertion mutagenesis of the wild-type *crp* gene with the Km^r cassette (containing the *aph3'-Ia* gene from pUT-Km1) was performed as described in the Supplemental Material. Upstream and downstream regions of the *crp* gene amplified are indicated. Restriction enzyme cleavage sites are indicated by vertical triangles. (**b**) PCR verified the insertion of the Km^r cassette, conferring kanamycin resistance, within the *crp* gene. PCR with I-out and argD-ovF primers was performed using the control plasmid pUTcrp-Km and chromosomal DNA from wild-type, *crp* mutant 1 or *crp* mutant 2 as a template. Primer binding sites are shown in **a**. The mutant DNA produced a 1486-bp PCR product but not in wild-type. M, 1-kb DNA marker; N, negative control; wt, wild-type. **(c)** Southern blot hybridization verified the *crp* mutant. Chromosomal DNA of wild-type, mutant 1 and mutant 2 was digested with

EcoRV and EcoRV/XhoI, respectively, before Southern blot hybridization using the labeled probe DNA indicated in **a**. EcoRV digestion resulted in a 3913-bp fragment in the wild-type but a 5227-bp fragment in mutants. EcoRV/XhoI digestion produced a 3913-bp fragment in the wild-type but a 2243-bp fragment in mutants. wt, wild-type. (**d**) Growth curves of the wild-type *P. mirabilis* in the absence or presence of glucose, the *crp* mutant and *crp*-complemented strain. The bacterial growth was expressed as the optical density at 600 nm (OD_{600}) on a $\log_{10}$ scale. Overnight bacterial cultures were diluted and grown to an OD_{600} of 0.01 and the growth was monitored at 1-h intervals. The data represent the averages and standard deviations of three independent experiments. wt, wild-type; crp, *crp* mutant; crpc, *crp*-complemented strain; wt-2%glc, wild-type with 2% glucose; wt-10%glc, wild-type with 10% glucose. (**e**) Correlation of gene expression between *crp* mutant and 10% glucose-treated wild-type *P. mirabilis*. Total RNA from wild-type (wt), 10% glucose-treated wild-type (wt-10%glc), or *crp* mutant (crp) was analyzed by RNA-seq. The x-axis indicates the $\log_2$ fold-change value in reads per kilobase per million (RPKM) of 10%glc-treated wild-type relative to the wild-type and the y-axis indicates the $\log_2$ fold-change value in RPKM of *crp* mutant relative to the wild-type. Each dot represents one gene.

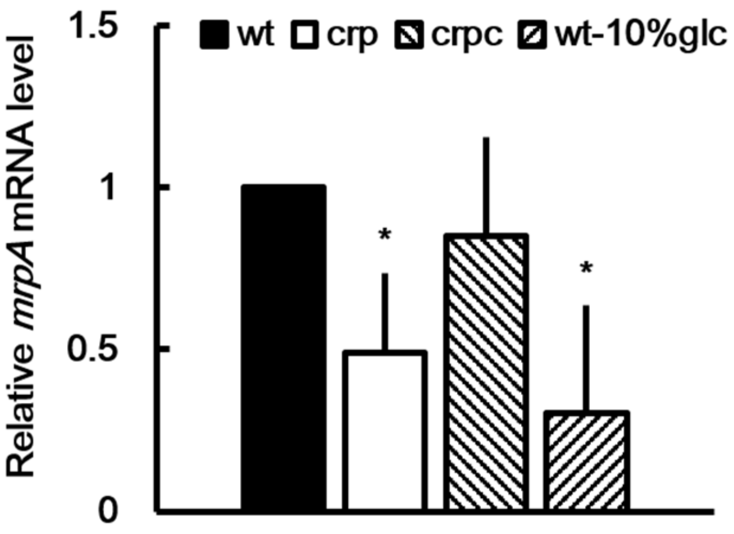

Supplementary Figure S2. Loss of *P. mirabilis crp* or high glucose affected *mrpA* expression. The *mrpA* mRNA amounts in the wild-type in the absence (wt) or presence of 10% glucose (wt-10%glc), *crp* mutant (crp), and *crp*-complemented strain (crpc) were quantified by real-time RT-PCR using bacterial cultures after incubation for 24 h. The value obtained for the wild-type cells was set at 1. The data represent the averages and standard deviations of three independent experiments. The significant difference from the wild-type is indicated with the asterisk (*, $P<0.05$ by Student's *t* test).

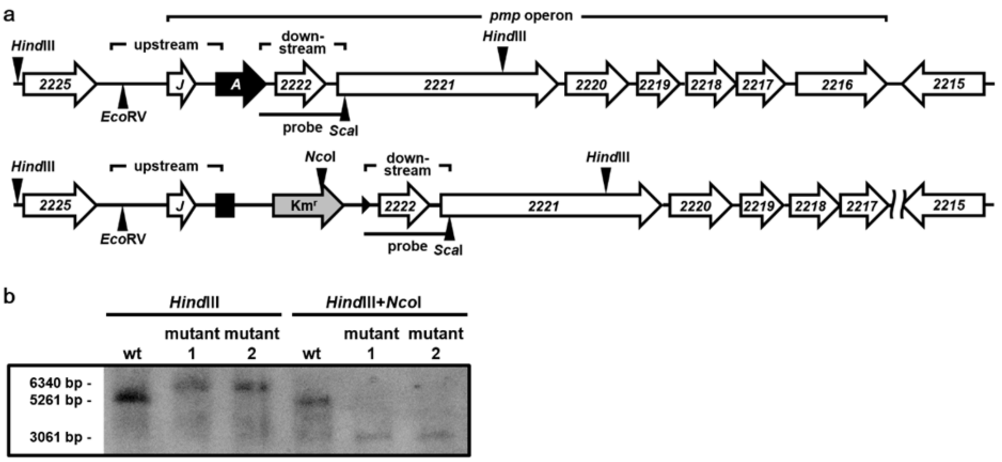

Supplementary Figure S3. Construction and verification of *P. mirabilis pmpA* mutant. (**a**) Insertion mutagenesis of the wild-type *pmpA* gene with the Km^{r} cassette (containing the *aph3'-IIa* gene from pKD4) was performed as described in the Supplemental Material. Upstream and downstream regions of the *pmpA* gene amplified are indicated. Restriction enzyme cleavage sites are indicated by vertical triangles. (**b**) Southern blot hybridization verified the insertion of the Km^{r} cassette within the *pmpA* gene. Chromosomal DNA of the wild-type, mutant 1 and mutant 2 was digested with HindIII and HindIII/NcoI, respectively, before Southern blot hybridization using the labeled probe DNA indicated in **a**. HindIII digetion resulted in a 5261-bp fragment in the wild-type but a 6340-bp fragment in mutants. HindIII/NcoI digestion produced a 5261-bp fragment in the wild-type but a 3061-bp fragment in mutants. wt, wild-type.

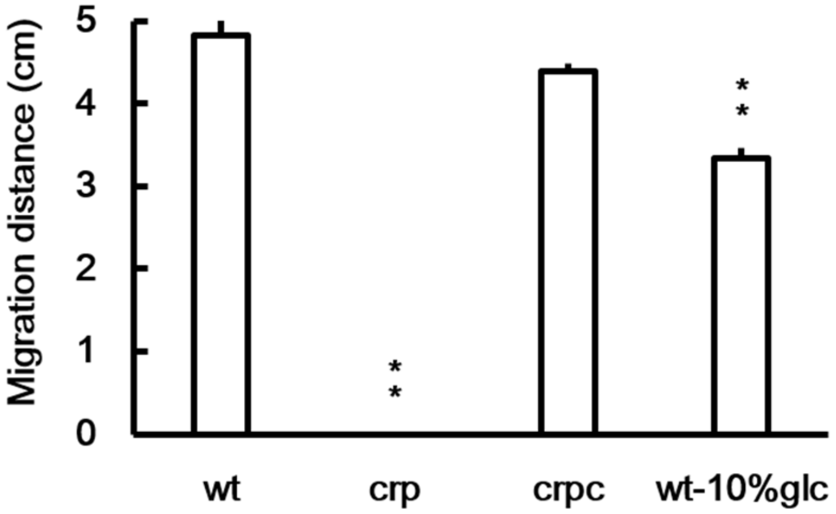

Supplementary Figure S4. Swimming migration of the wild-type *P. mirabilis* in the absence or presence of 10% glucose, *crp* mutant and the *crp*-complemented strain. Overnight cultures were inoculated centrally into the swimming agar (0.3%, w/v) plates. The migration distance was measured at 16 h after inoculation. The data represent the averages and standard deviations of three independent experiments. The significant difference from the wild-type is indicated with the asterisk (**, *P*<0.01 by Student's *t* test). wt, wild-type; wt-10%glc, wild-type with 10% glucose; crp, *crp* mutant; crpc, *crp*-complemented strain.

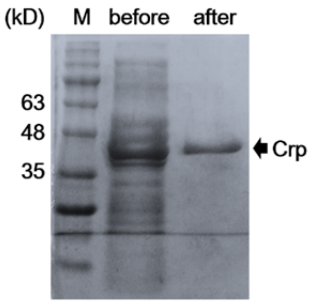

Supplementary Figure S5. The His-tagged recombinant Crp protein before and after purification. *E. coli* BL21(DE3) cells carrying the plasmid for expression of His-tagged recombinant Crp were induced by 0.1 mM IPTG for 3 h at 25°C, the cell pellet was collected and total proteins were extracted by sonication and centrifugation. The soluble fraction was analyzed by 12% SDS-PAGE before and after being purified by a Ni^{2+}-nitrilotriacetic acid column. M, protein ladder.

ptsG

E. coli MG1655

TATTGTGACATATGTTTTGTCAAAATGTGCAACTTCTCCAATGATCTGAAGTTGAAACGTGATAGCCGTCAAACAAATTGGCACTG
AATTATTTTACTCTGTGTAATAAATAAAGGGCGCTTAGATGCCCTGTACACGGCGAGGCTCTCCCCCCTTGCCACGCGTGAGAAC
GTAAAAAAAGCACCCATACTCAGGAGCACTCTCAATT**ATG** *ptsG* translation start site

P. mirabilis N2

AAACGTGACAGCTATCATATATTCAATAATGGATTTATTTTATGATGTAAAATAAATAGAGCTTATTATTAGATAAGTTAATTGAATAA
ACCTACCTTAACTTTAAACAAATAAAAAACTTATAACACATACAACCCATATTCGGGAGTCTGCTTG**ATG** *ptsG* translation start site

crp

E. coli MG1655

AAGGCGACCTGGGTCATGCTGAAGCGAGACACCAGGAGACACAAAGCGAAAGCTATGCTAAAACAGTCAGGATGCTACAGTAAT
ACATTGATGTACTGCATGTATGCAAAGGACGTCACATTACCGTGCAGTACAGTTGATAGCCCCTTCCCAGGTAGCGGGAAGCATA
TTTCGGCAATCCAGAGACAGCGGCGTTATCTGGCTCTGGAGAAAGCTTATAACAGAGGATAACCGCGC**ATG** *crp* translation start site

P. mirabilis N2

TATTATAATCTATTCCTTATTAACTTTCCGAATTCTCAGGAAGTTAATCGGTAAAGATAGGATTTTAAGCACGCTGTATCAAAAGGG
TGTCAATCCGTTTTGAAAGGCTGCATTAACGTACAGAGGATAACGCGA**ATG** *crp* translation start site

cyaA

E. coli MG1655

TTTACGGTCAATCAGCAAGGTGTTAAATTGATCACGTTTTAGACCATTTTTTCGTCGTGAAACTAAAAAAACCAGGCGCGAAAAGT
GGTAACGGTTACCTTTGACATACGAAATATCCCGAATGCCGCGTGTTACCGTTGATGTTGGCGGAATCACAGTCATGACGGGTAG
CAAATCAGGCGATACGTC**TTG** *cyaA* translation start site

P. mirabilis N2

TTTACGCCTTGCTTGCAAGGTGTTAAATTGATCACGTTTCCGAGATTTTGTTAGTAAAGTTTTATCTATTAATAATTGAATAATTAAA
GCTCGGTAATTTATAAACTCAGATTCTACTATTTTCTTAGTATTGTTCAGGCGAAACTC**TTG** *cyaA* translation start site

hfq

E. coli MG1655

GGTTGGGAAGGGGTTCACTGGCTTGACAGTGAAAAACCAGAACAGGCGCGTGACGAAGTATTACAGGTTGTTGGTGCTATCGCA
GGCTGAATGTGTACAATTGAGACGTATCGTGCGCAATTTTTTCAGAATCGAAAGGTTCAAAGTACAAATAAGCATATAAGGAAAAG
AGAGA**ATG** *hfq* translation start site

P. mirabilis N2

GGTTGGCATGATGTTCATTGGTTAGATAGTGAAGATTTTGAACAGTCTCTTAACACTGTTTTGCAGGTAGTTAGTGCATAGATGGT
AGGTTTGTGTAAAATTGATAGTTGCTAAAGTGCAATTTTTGAGTAGTTCATTTTTTCGAACCATTAGGTTCTAGTATAAAACAACAAA
ATAAGGAAAACATAGA**ATG** *hfq* translation start site

Supplementary Figure S6. The upstream regulatory regions of *ptsG*, *crp*, *cyaA* and *hfq* of *P. mirabilis* N2 and *E. coli* MG1655 showing the putative Crp-cAMP binding sites. Putative Crp-cAMP binding sites are underlined. The -10 and -35 boxes or the transcription start site of the sigma 70 promoter is framed.

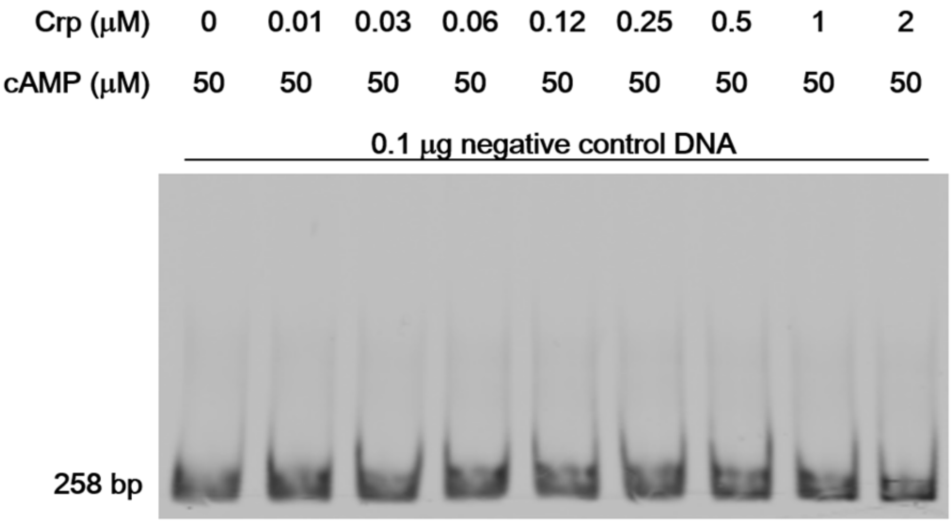

Supplementary Figure S7. Electrophoretic mobility shift assay of His-tagged Crp and IRDye-labeled negative control DNA fragment. The 258-bp negative control DNA fragment was amplified from the pGEM-T easy using the IRDye-labeled M13F/M13R primers. The DNA product (0.1 μg) was incubated with the indicated concentrations (0-2 μM) of Crp protein in the presence of cAMP. After protein-DNA complex formation, the fragments were resolved on a 5% non-denaturing polyacrylamide gel and the gel image was obtained by the quantitative infrared fluorescent imaging system.

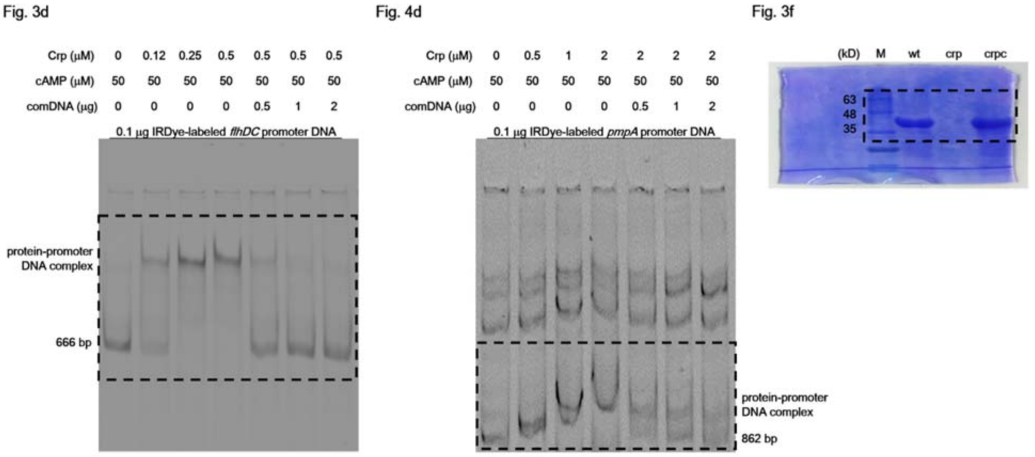

Supplementary Figure S8. Full-length images of gels presented in the main figures 3d, 3f and 4d.

Supplementary Table S1. Bacterial strains and plasmids used in this study.

Strain or plasmid	Genotype or relevant phenotype	Reference
P. mirabilis		
wt	wild-type N2; Tet^r	Clinical isolate
crp	wt derivative; *crp* knockout mutant; Km^r	This study
crpc	*crp* mutant containing pGcrp-com; *crp*-complemented strain; Amp^r	This study
hfq	wt derivative; *hfq* knockout mutant; Km^r	1
pmpA	wt derivative; *pmpA* knockout mutant; Km^r	This study
pmpA/crp	wt derivative; *pmpA/crp* double mutant; Km^r Gm^r	This study
rpoS	wt derivative; *rpoS* knockout mutant; Km^r	1
rpoSc	*rpoS* mutant containing pGrpoS-com; *rpoS*-complemented strain; Amp^r	1
E. coli		
DH5α	*fhuA2 lac(del)U169 phoA glnV44 thi-1 hsdR17 Φ80' lacZ(del)M15 gyrA96 recA1 relA1 endA1*	Invitrogen
S17-1 λ *pir*	λ *pir* lysogen of S17-1 [*thi pro hsdR*$^-$ *hsdM*$^+$ *recA* RP4 2-Tc::Mu-Km::Tn*7* (Tp^r Sm^r)]; permissive host able to transfer suicide plasmids requiring the Pir protein by conjugation to recipient cells	Biomedical
BL21(DE3)	F$^-$ *ompT hsdS*$_B$ (r_B^- m_B^-) *gal dcm* (DE3)	Invitrogen
Plasmids		
pGEM-T Easy	High-copy TA cloning vector; Amp^r	Promega
pUT-Km1	Suicide plasmid requiring the Pir protein for replication and containing a mini-Tn*5* cassette containing Km^r gene	2
pKD4	pKD4 is a template plasmid for frt-flanked Km^r cassette. The Km^r cassette originally came from pCP15.	3
pX1918GT	A *xylE*-Gm^r fusion cassettes-containing plasmid	4
pET32a(+)	Expression vector containing T7 promoter; Amp^r	Novagen

Tet, tetracycline; Amp, ampicillin; Gm, gentamicin; Km, kanamycin; Sm, streptomycin; Tp, trimethoprim.

Supplementary Table S2. Primers used in this study.

Primer	Sequence (5' to 3')	Description
crp-upF	GTCGACTATCAATACTATTGTTGTTCCAGG	For *crp* knockout, and *crp* reporter assay. Paired with "XbaI-crp-upR".
XbaI-crp-upR	TCTAGATTGTGAATATGGCAGTGTGA	
XbaI-crp-dnF	TCTAGACGTGAAACTGTAGGCCGTAT	For *crp* knockout. Paired with "crp-dnR".
crp-dnR	GCATGCGCATCCACTATTGAATAAAG	
I-out	GAGCTCGAATTCGGCCTAG	Check for *crp* knockout mutant. Paired with "argD-ovF".
argD-ovF	GTATAGCTAAATGTCGGTCAGGT	
crp-comF	AATTGTACAGCTCGCTCAAC	For *crp* complementation. Paired with "crp-comR".
crp-comR	GCATGGCTTACGTACACTAT	
pmpA-upF	GCATGCTTGAGATAGACACCAGCTAT	For *pmpA* knockout, and *pmpA* reporter assay. Paired with "XbaI-pmpA-upR".
XbaI-pmpA-upR	TCTAGAAGGCTTTTGCTGATTTGACCA	
XbaI-pmpA-dnF	TCTAGACTGAAGGTGAGTTTAACGCT	For *pmpA* knockout. Paired with "pmpA-dnR".
pmpA-dnR	GTCGACTTGATGAACCGATCCCTTGT	
FRT-out	CCTATTCCGAAGTTCCTATTC	Check for *pmpA* knockout mutant. Paired with "pmpA-upF".
xylE-R	AAGTCGTACCGGACCATCAG	Check for *pmpA/crp* double mutant. Paired with "argD-ovF".
flhDC-reF	GGGTAGATTCGCTTATTAATTCTC	For *flhD* reporter assay and EMSA. Paired with "flhDC-reR".
flhDC-reR	CTCTTTACATCCCGTCCGAT	
crp-purF	GGATCCATGGTTCTCGGCAAGCCGCAAA	For Crp-His_6 expression. Paired with "crp-purR".
crp-purR	CTCGAGTTAACGAGTACCGTAAACAACG	
ptsG-rtF	CGCGAAACAAGCGGATAAA	For *ptsG* real-time RT-PCR. Paired with "ptsG-rtR".
ptsG-rtR	CGCAAGCGTGTAATACAAGCA	
crp-rtF	CCGCCAATTAATTCAGGTCAA	For *crp* real-time RT-PCR. Paired with "crp-rtR".
crp-rtR	GCGTCATTGCATCAGGTTGTT	
hfq-rtF	ACGGCATTAAATTACAGGGTC	For *hfq* real-time RT-PCR.

	AGA	Paired with “hfq-rtR”.
hfq-rtR	GCTATGATGAGAAACAGGACGAGAA	
cyaA-rtF	TTGCATCGATGAGTGATGTG	For *cyaA* real-time RT-PCR. Paired with “cyaA-rtR”.
cyaA-rtR	TATTGACCCATTGACGTTGC	
flhDC-rtF	CACGAGCATGGACATTAG	For *flhDC* real-time RT-PCR. Paired with “flhDC-rtR”.
flhDC-rtR	GCAGGATTGGCGGAAAGTT	
mrpA-rtF	GGTTCTTTAGGCATTGAAGG	For *mrpA* real-time RT-PCR. Paired with “mrpA-rtR”.
mrpA-rtR	TCATTGTTACCATCACGCAG	
rpoS-rtF	GCCTTATTCGTGCTGTTG	For *rpoS* real-time RT-PCR. Paired with “rpoS-rtR”.
rpoS-rtR	GACGAATAGTGCGGGTTT	
pmpA-rtF	AATGGCTTTTCTGCGACTGTT	For *pmpA* real-time RT-PCR. Paired with “pmpA-rtR”.
pmpA-rtR	TTGGTTAGCACTTTCGGTAGCA	
gfpuv-rtF	TTCAATGCTTTTCCCGTTATCC	For *gfpuv* real-time RT-PCR. Paired with “gfpuv-rtR”.
gfpuv-rtR	GCGTCTTGTAGTTCCCGTCATC	
gyrB-rtF	GACCCGTACGCTAAACAAC	Internal control for real-time RT-PCR. Paired with “gyrB-rtR”.
gyrB-rtR	AGAAATAACCGCAATCAGG	
pmp-fpF	TACGTAAATCTCTAACAGGAGCC	Amplification of *pmpA* promoter for DNase I footprinting and EMSA. Paired with “pmp-koR”.
pmp-koR	AGGCTTTTGCTGATTTGACCA	
M13F	CACGACGTTGTAAAACGAC	M13 primers labelled with IRDye or not for EMSA. Paired with “M13R”.
M13R	GGATAACAATTTCACACAGG	

The recognition sequences of endonucleases are indicated.

Supplementary Methods

Construction of *P. mirabilis* mutants and *crp*-complemented strain. Sequences flanking the *crp* gene was amplified by PCR using the primer pairs crp-upF/XbaI-crp-upR and XbaI-crp-dnF/crp-dnR, respectively for construction of *crp* mutant, and cloned into pGEM-T Easy (Promega) to generate pGcrp-up and pGcrp-dn. pGcrp-up was digested with SalI/XbaI, and the *crp* upstream sequence-containing fragment was ligated to SalI/XbaI-digested pGcrp-dn to produce the pGcrp-updn plasmid, which contains both upstream and downstream sequences of *crp*. A Km^{r} cassette was inserted in the XbaI-digested pGcrp-updn plasmid to generate pGcrp-updn-Km. For *pmpA* mutant, pGpmpA-updn-Km was constructed in a similar way except using primer pairs pmpA-upF/XbaI-pmpA-upR and XbaI-pmpA-dF/pmpA-dnR. The DNA fragment containing the Km^{r} cassette-disrupted combined upstream and downstream sequences of *crp* or *pmpA* was cleaved by SalI/SphI from pGcrp-updn-Km or pGpmpA-updn-Km, and ligated into SalI/SphI-cleaved pUT-Km1 to generate pUTcrp-Km and pUTpmpA-Km, respectively. The *pmpA*/*crp* double mutant (Km^{r} Gm^{r}) was constructed in a similar way using an existing *pmpA* mutant (Km^{r}). A *xylE*/Gm^{r} cassette was inserted in the XbaI-digested pUTcrp-Km plasmid for the *pmpA*/*crp* double mutant to generate pUTcrp-*xylE*/Gm. Gene inactivation mutagenesis by homologous recombination and

confirmation of mutants with double-crossover events by colony PCR and Southern blot hybridization were performed (Supplementary Fig. S1a, b, c, and Supplementary Fig. S3) as described previously[2].

For complementation of *crp* mutant, the fragment containing full-length *crp* gene was amplified by PCR using the primer pair crp-comF/crp-comR, and cloned into pGEM-T Easy to generate the plasmid pGcrp-com. *crp* is thus driven by its own promoter. The plasmid pGcrp-com was then transformed into the *crp* mutant to generate the *crp*-complemented strain.

Growth curve analysis. Bacteria were grown overnight at 37°C, the cultures were diluted to an initial optical density at 600 nm (OD_{600}) of 0.01 and then the OD_{600} values were measured at 1-h intervals up to 24 h with a spectrophotometer.

Swimming assay. The swimming migration was determined on 0.3% (w/v) LB agar plates with 10% glucose or not after incubation for 16 h at 37°C.

Nucleotide sequence accession number. The nucleotide sequences of *P. mirabilis crp* gene have been deposited in GenBank under the accession number KP278143.

Supplementary References

1. Wang, M. C., Chien, H. F., Tsai, Y. L., Liu, M. C. & Liaw, S. J. The RNA chaperone Hfq is involved in stress tolerance and virulence in uropathogenic *Proteus mirabilis*. *PLoS One* **9,** e85626; 10.1371/journal.pone.0085626 (2014).
2. Liu, M. C., Kuo, K. T., Chien, H. F., Tsai, Y. L. & Liaw, S. J. New aspects of RpoE in uropathogenic *Proteus mirabilis*. *Infect. Immun.* **83,** 966-977 (2015).
3. Datsenko, K. A. & Wanner, B. L. One-step inactivation of chromosomal genes in *Escherichia coli* K-12 using PCR products. *Proc. Natl. Acad. Sci. U. S. A.* **97,** 6640-6645 (2000).
4. Schweizer, H. P. & Hoang, T. T. An improved system for gene replacement and *xylE* fusion analysis in *Pseudomonas aeruginosa*. *Gene* **158,** 15-22 (1995).
